# Human Rotavirus VP6-Specific Antibodies Mediate Intracellular Neutralization by Binding to a Quaternary Structure in the Transcriptional Pore

**DOI:** 10.1371/journal.pone.0061101

**Published:** 2013-05-09

**Authors:** Mohammed S. Aiyegbo, Gopal Sapparapu, Benjamin W. Spiller, Ilyas M. Eli, Dewight R. Williams, Robert Kim, David E. Lee, Tong Liu, Sheng Li, Virgil L. Woods, David P. Nannemann, Jens Meiler, Phoebe L. Stewart, James E. Crowe

**Affiliations:** 1 Department of Pathology, Microbiology and Immunology, Vanderbilt Medical Center, Nashville, Tennessee, United States of America; 2 Department of Pediatrics, Vanderbilt Medical Center, Nashville, Tennessee, United States of America; 3 Department of Molecular Physiology and Biophysics, Vanderbilt Medical Center, Nashville, Tennessee, United States of America; 4 The Vanderbilt Vaccine Center of Vanderbilt Medical Center, Nashville, Tennessee, United States of America; 5 Department of Chemistry, Vanderbilt University, Nashville, Tennessee, United States of America; 6 School of Medicine, University of California San Diego, La Jolla, California, United States of America; Nanyang Technological University, Singapore

## Abstract

Several live attenuated rotavirus (RV) vaccines have been licensed, but the mechanisms of protective immunity are still poorly understood. The most frequent human B cell response is directed to the internal protein VP6 on the surface of double-layered particles, which is normally exposed only in the intracellular environment. Here, we show that the canonical VP6 antibodies secreted by humans bind to such particles and inhibit viral transcription. Polymeric IgA RV antibodies mediated an inhibitory effect against virus replication inside cells during IgA transcytosis. We defined the recognition site on VP6 as a quaternary epitope containing a high density of charged residues. RV human mAbs appear to bind to a negatively-charged patch on the surface of the Type I channel in the transcriptionally active particle, and they sterically block the channel. This unique mucosal mechanism of viral neutralization, which is not apparent from conventional immunoassays, may contribute significantly to human immunity to RV.

## Introduction

Rotaviruses, double-stranded RNA viruses that belong to the *Reoviridae* family, are the major causative agents for acute gastroenteritis in infants and young children worldwide [1]. Almost all children are infected with rotavirus (RV) by age 5, and infection results in an estimated half million deaths each year in children younger than 5 years of age [2]. The RV genome consists of 11 segments of double-stranded RNA that each code for a single protein, with the exception of segment 11 that codes for two proteins. The virions are non-enveloped, triple-layered, icosahedral viruses. The triple-layered particle (TLP) is composed of an inner capsid layer of virus protein 2 (VP2) protein, an intermediate capsid layer of VP6, and an outer capsid layer made up of VP7 and intermittent spikes of VP4 protein [3]–[7].

The intermediate and outer capsid layers both have a T = 13 *l* icosahedral symmetry that defines 132 channels within the viral architecture into three types based on their position with respect to the T = 13 icosahedral symmetry axis [6], [8]–[11]. There are 12 Type I channels located at the icosahedral five-fold axes that have narrow openings through which nascent viral mRNA egresses out of the particle during viral transcription [4]. The Type II channels located at the quasi-six-fold axes directly adjacent to the Type I channels have larger openings than the Type I channels. The Type III channels also have larger openings than the Type I channels and are located at the quasi-six-fold axes not directly adjacent to the Type I channels and close to the icosahedral three-fold axes. RV, in its TLP form, is transcriptionally-inactive; the double-layered particle (DLP) is transcriptionally-active [7], [12]–[14]. The viral transcription machinery, composed of VP1 and VP3, is located near the icosahedral five-fold axis below the VP2 layer [4]. In the TLP, VP7 obstructs the Type I channels located at the five-fold axes, causing a steric hindrance that blocks the exit of nascent mRNAs [15]. VP7 also induces a global conformational change in the VP6 trimer arrangement that results in a narrowing of the Type I channels at the five-fold axes as observed in the high-resolution cryo-EM structure of the DLP recoated with recombinant VP7 [13]. The fact that VP7 changes the orientation of the VP6 timers around the Type I channel also was noted in an earlier moderate resolution cryo-EM study comparing VP2–VP6 with VP2–VP6–VP7 recombinant particles [16].

Protective and neutralizing antibodies induced in animals or humans following a rotavirus infection classically are thought to be directed against VP4 and VP7 [17], [18]. However, the highest serum titers of human antibodies binding to RV after infections typically are directed against VP6. Our laboratory previously identified the molecular basis for the natural human B cell response to RV VP6, comprising an antibody repertoire that is dominated by the use of a single antibody heavy chain variable gene, V_H_1-46 [19]. VP6-specific human antibodies encoded by V_H_1-46 are the most common RV-specific antibodies in B cells made by infants and adults, including intestinal homing B cells [19]–[22].

It is possible that VP6 antibodies simply represent a common response to highly antigenic features on degraded viral particles or infected cell debris and do not contribute to RV immunity. Recent animal model studies, however, suggested that VP6-specific antibodies might play a role in immunity to RV. Some murine VP6-specific antibodies of the IgA isotype, which do not neutralize virus in conventional *in-vitro* neutralization assays, protect mice from RV infection and clear chronic RV infection in SCID mice [23]. Subsequent *in vitro* studies in polarized epithelial cells showed that the murine anti-VP6 IgA monoclonal antibody 7D9 inhibited RV replication inside epithelial cells at an early stage of infection [24], which later was shown to depend on transcytosis of dimeric IgA mediated by the polymeric immunoglobulin receptor (pIgR) [25]. We sought to determine if the dominant type of human humoral response to RV infection comprising V_H_1-46 germline gene-encoded antibodies directed against VP6 plays a functional role in inhibiting virus. Such activity should be of high interest, since the correlates of protective immunity to RV in humans are poorly understood, and the response to VP6 is common in humans.

## Results

### Generation and characterization of recombinant dimeric IgA

**Figure 1:**
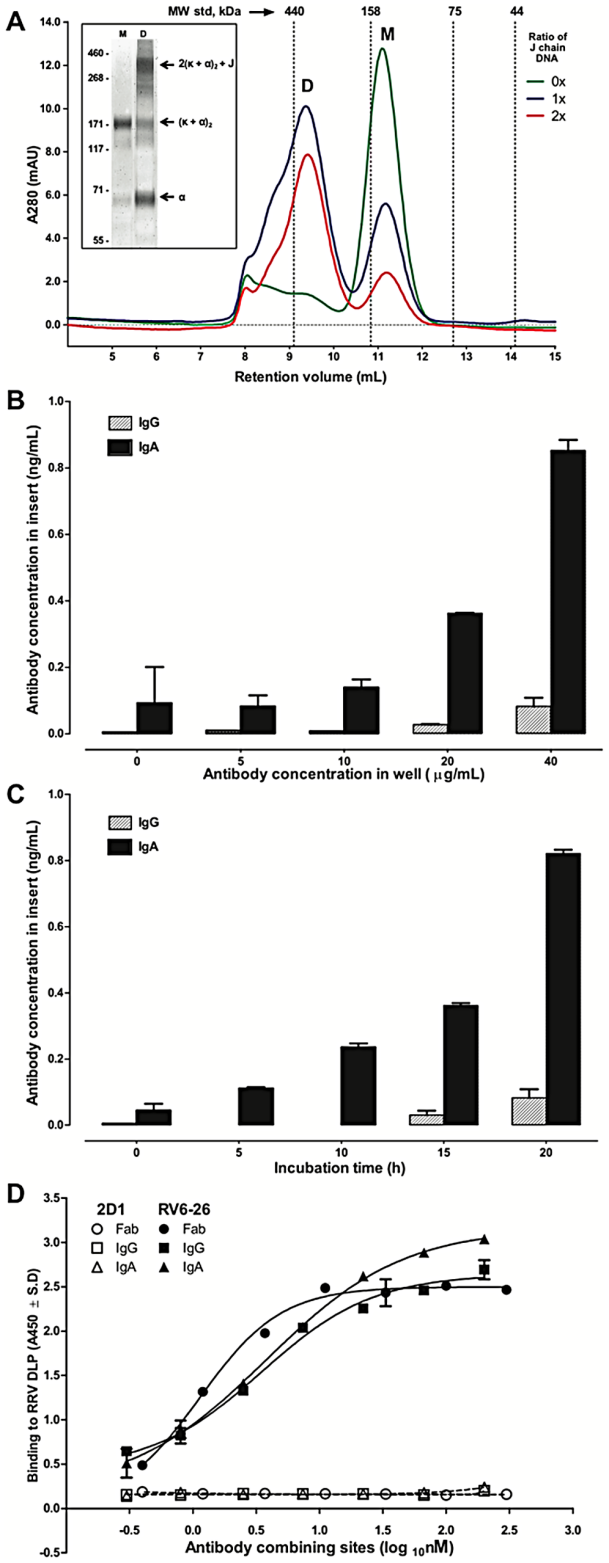
Recombinant IgA expression, assembly and function. (A) Transient expression of dimeric IgA was done in 293F cells using a mixture of α, κ and varying amounts of J chain plasmid DNA. Antibody purified from the culture supernatant was fractionated on a Superdex 200 column to monitor the assembly and the different traces represent the amounts of J chain DNA used in transfection (green – 0; blue – 1 and red – 2 times (0×, 1×, or 2×) the amount of α chain DNA). Molecular weight marker standards, as shown, were used for constructing a standard curve. Peaks indicating dimer (D) or monomer (M) fractions are marked. Inset: SDS-PAGE under non-reducing conditions and immunoblotting of dimer and monomer fractions with anti-α chain antibody. Different molecular species are labeled. These data were obtained as aliquots from different fractions from the size exclusion column, and we used them at the concentrations at which they were obtained. Some differences in the relative amounts of one band to another also can be attributed to consequences of electrophoresis conditions (boiling in the presence of denaturing agent). (B) Dose response of IgG or dimeric IgA transcytosis across a polarized epithelial monolayer was tested by adding antibodies to the bottom compartment at different concentrations and measuring the concentration in the supernatant of Transwell inserts after 22 h incubation. (C) Time-course of IgA transcytosis was measured similarly by adding 40 µg/mL antibodies in the bottom compartment and collecting the supernatant in Transwell inserts at indicated time points. (D) RRV DLP were coated on microplates and differing concentrations of Fab, IgG or IgA forms of RV6-26 or 2D1 control human antibody (specific for the HA protein of 1918 influenza) normalized for binding sites (Fab = 1; IgG = 2 and IgA = 4) were allowed to bind to DLP. Bound antibodies were detected using peroxidase-conjugated anti-human κ chain antibodies, and the absorbance values are shown.

We previously isolated human anti-VP6 antibodies and expressed these as Fabs in a bacterial expression system [26]. In the current study, we sought to test the activity of these antibodies as dimeric IgA molecules in a model of immunity at mucosal epithelia. RV6-26 and a control antibody were expressed as recombinant IgG or IgA molecules. Assembly of dimeric IgA requires the presence of joining (J) chain in addition to α and κ chains. To determine the optimal amount of J chain necessary for efficient formation of dimeric IgA, different ratios of J chain and α chain DNA were tested in replicate transfections, and the antibodies harvested from culture supernatant were purified on anti-IgA agarose. Purified IgA antibodies were resolved on a size exclusion column, and the amounts of monomeric or dimeric IgA were determined by calculating the area under the curve for each fraction. We found that a 1∶2 ratio of α to J chain DNA enabled the formation of the highest proportion of dimers (Figure 1A), and); higher amounts of J chain did not offer any additional benefit in dimerization efficiency (data not shown). We confirmed the presence of monomeric or dimeric IgAs in fractions using electrophoresis under non-reducing conditions followed by immunoblotting with anti-α chain antibodies (Figure 1A, inset).

The IgA antibodies then were tested for the ability to transcytose across a polarized epithelial monolayer. MDCK cells stably expressing human pIgR were grown on Transwell inserts, and the presence of a tight monolayer indicating a high level of polarity was verified by measuring transepithelial resistance to be above 280 Ω.cm^2^ before and after antibody incubation. The polymeric IgA form of RV6-26, but not IgG form, displayed dose- and time-dependent transcytosis across the polarized epithelial cell monolayer (Figure 1B and C). Transcytosis experiments also were conducted with polarized Caco-2 cells, with similar results (data not shown).

Molecular engineering of the constant domains of antibodies can sometimes lead to change in function of the antigen-binding domain [27], [28]. To confirm that the IgG and IgA forms retained similar antigen binding affinity as the parent Fab, we compared binding of the various antibody forms to DLPs coated on ELISA plates. IgG and IgA forms of RV6-26 displayed similar binding characteristics as the parent Fab molecule (Figure 1D) [26].

### MAb RV6-26 inhibits mRNA transcription from DLP

**Figure 2:**
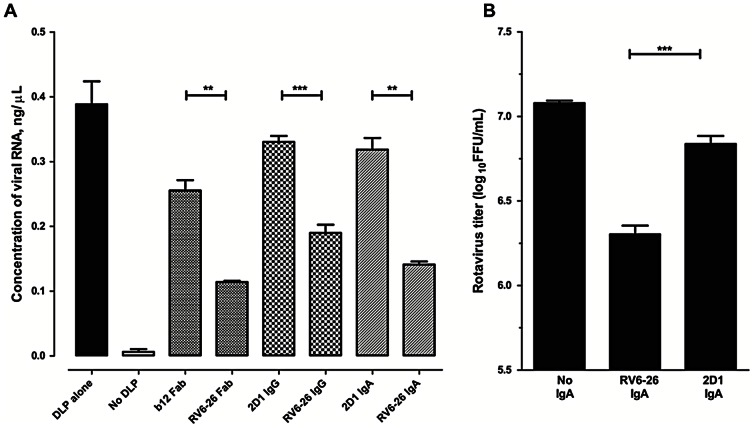
Anti-rotaviral activity of RV6-26. (A) Inhibition of *in vitro* transcription: EDTA-activated DLP were incubated with 200 nM combining sites of different antibodies and mRNA was synthesized *in vitro* using selected components of the Riboprobe SP6 system (transcription was mediated by the viral RNA-dependent RNA polymerase not the SP6 DNA-dependent RNA polymerase). First-strand cDNA was synthesized by reverse transcription using a VP6-specific primer. Amplification of cDNA with VP6-specific primers was monitored in a real-time PCR using SYBR Green; the concentrations of RNA estimated from a standard curve constructed using reference RNA extracted from RRV are plotted. (B) Inhibition of rotavirus replication by IgA: polarized monolayers of Caco-2 cells grown on Transwell inserts were treated with polymeric IgA in the basal compartment and inoculated apically with trypsin-activated RRV (MOI = 5) at ambient temperature for 1 h and then cultured for 16 in medium containing trypsin. Amount of rotavirus in the inserts was titrated by inoculating MA104 cells and culturing for 16 h, followed by acetone-fixation and staining with anti-rotavirus polyclonal antibodies conjugated to either Alexa568 or IRDye 800. Detection was done either by scanning on Licor or by counting fluorescent foci.

The intact, extracellular form of RV is a triple-layered particle that is transcriptionally inactive, while the DLP found in the intracellular compartment is transcriptionally active. We tested the hypothesis that RV6-26 reduces viral replication by inhibiting the efficiency of transcription by DLPs. We measured the effect of RV6-26 in the form of an Fab, IgG or dimeric IgA molecule on the *in vitro* production of mRNA from DLP. We transcribed mRNA from EDTA-activated DLPs in the presence of mAb RV6-26 or control antibodies and quantified the amount of mRNA using either a quantitative PCR assay (Figure 2A) or by measuring the amount of incorporation of radiolabeled CTP during *in vitro* transcription by DLPs (data not shown). Each of the three forms of RV6-26 inhibited transcription from DLPs, compared to the control antibodies.

### Intracellular neutralization of RV by RV6-26 polymeric IgA during transcytosis

Since RV6-26 inhibited transcription *in vitro*, the next step was to determine if the antibody could affect viral replication in cells using the physiologic route of interaction inside cells, which is mediated by transcytosis of IgA. We tested the activity of the dimeric IgA form of RV6-26 in neutralizing RV in polarized Caco-2 intestinal cell line culture monolayers grown on Transwell inserts. Caco-2 cells were inoculated into Transwell inserts and incubated for about 21 days to achieve a tight, polarized monolayer. The cultures were treated with RV6-26 dimeric IgA or a control IgA on the basolateral surface of the cells for 4–6 h prior to inoculation of cells with trypsin-activated RV on the apical surface. The amount of virus in the supernatant of the individual inserts was quantified, as described in the Methods. RV6-26 IgA reduced the viral titers in the inserts compared to the control IgA (Figure 2B). The reduction was modest but reproducible. Taken together with the *in vitro* transcription data, these observations suggest strongly that the neutralizing activity of RV6-26 is a consequence of the inhibition of transcription inside cells during basolateral-to-apical transcytosis of RV-specific IgA.

### RV6-26 binds to VP6 molecules in the Type I, II, and III channels on DLPs

**Figure 3:**
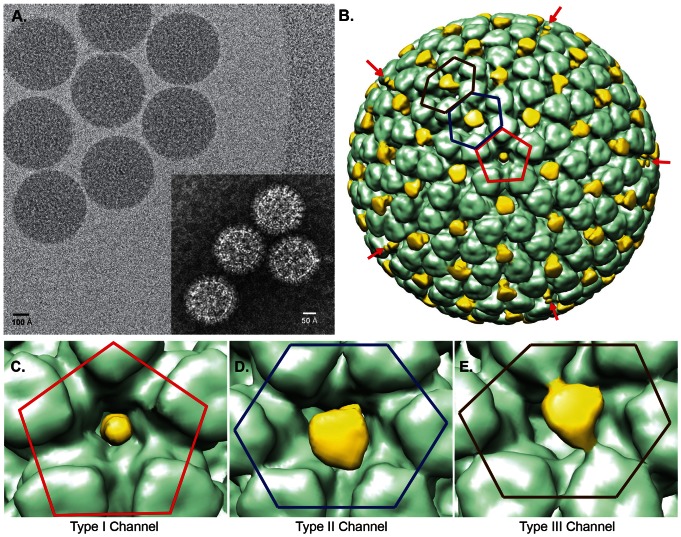
Structure of RV6-26 Fab bound to RV-DLP. (A) A representative cryo-micrograph of RV6-26-DLP complexes vitrified in ice over a hole in Quantifoil holey carbon grids. The white boxes indicate complexed particles that were extracted and processed. The inset shows a representative cryo-micrograph of unbound RV-DLP. (B) Surface representation of the resulting 3D structure of RV6-26-DLP complex reconstructed to a 10.9 Å resolution. RV6-26 Fab (yellow) at five-fold axis is indicated by red pentagon (also shown in [C]) Red arrows indicate the location of additional five-fold axes on the structure. The Fabs bound in the pseudo-six-fold axes directly adjacent to the transcriptional pores (blue hexagon [D]) exhibit a different average density representation from those bound at the pseudo-six-fold axes not directly adjacent to the transcriptional pore (brown hexagon [E]).

It was of interest to determine the pattern of binding of RV6-26 to DLPs, in order to begin to determine the specific mechanism of action. RNA transcription occurs at the base of the Type I channel, which is located at the icosahedral five-fold symmetry of the VP6 layer. The VP1 and VP3 transcription complex assembles on the VP2 core at each of the five-fold axes of the virion particle. We sought to determine if RV6-26 bound to DLPs at this channel, and if so, to define the mode of binding. We determined the structure of complexes of RV6-26 Fab and RV DLPs using cryo-electron microscopy (cryo-EM) reconstruction; data is deposited in the Electron Microscopy Data Bank (EMDB ID *pending*). The reconstruction of the Fab-DLP complex was computed from 3,029 complex particle images extracted from corresponding cryo-EM micrographs; a representative micrograph is shown in Figure 3A. Numerous cryo-EM structures have been determined for icosahedral viral particles in complex with antibodies and receptors [29]. Normally, the antibody or receptor extends away from the viral surface and is visible in cryo-electron micrographs. Even Fab fragments bound to viral particles typically are visible in the cryo-electron micrographs [30], [31]. In the case of the Fab-DLP complex, the cryo-electron micrographs revealed that the particles were smooth without any projecting Fab density. After image reconstruction the estimated final resolution for the Fab-DLP complex was 10.9 Å, using the 0.5 FSC criterion. Consistent with the cryo-electron micrographs, the structure also was relatively smooth with the Fab density situated between VP6 trimers rather than protruding from the outer surface of the viral particle (Figure 3B).

The structure revealed a number of interesting features of binding. Type I, II, and III channels of the DLP clearly were occupied by the Fab. Interpretation of the data was complicated, as reconstructing the density map from 3,029 particles averaged the density of Fabs bound to these channels. For example, we observed Fab density only in the center of the channel, not directly touching the DLP. Further, the Fab density was much smaller than the expected size of an Fab - approximately only 14, 34 and 31% for channel Type I, II and III, respectively. We attribute this finding to the fact that only one Fab can fit into each channel at any given point in time. If one Fab is bound in each channel and this binding is not preferential toward any one VP6 trimer, then the contribution of the Fab would be averaged due to the imposed icosahedral symmetry in differing ways in the Type I, II or III channels. Density at the Fab-DLP interface is expected to be weakened to 20% (Type I channel) and 17% (Type II and Type III channels) and to be present at 100% only where Fabs binding to different trimers overlapped. For Type I channels a perfectly five-fold symmetric Fab density was observed, because the Type I channel is located at the five-fold axis (Figure 3C), and icosahedral symmetry is imposed in data processing. Asymmetric Fab reconstructions were observed at the quasi-six fold axis (the Type II and Type III channels; Figure 3D, F). This asymmetry can be explained by the fact that these channels are not perfectly symmetric but locate on the quasi six-fold axis. If the hexagonal channel is deformed in one dimension, Fabs will overlap more extensively, leading to additional density in these areas. Alternatively, preferential binding or differential accessibility could explain the asymmetry. In the Type III channels, additional density links the Fab to the trimers, which are adjacent to the five-fold trimers and icosahedral three-fold trimers, respectively (Figures 3B and 3E). However, a model assuming equal probability for all Fab-trimer interactions (see below) suggests that no preferential binding or differential accessibility is needed to explain the experimental results. The observed reduction in density for Fab was consistent with a single Fab binding in a non-preferential manner per channel.

### RV6-26 binds to a quaternary epitope on VP6

**Figure 4:**
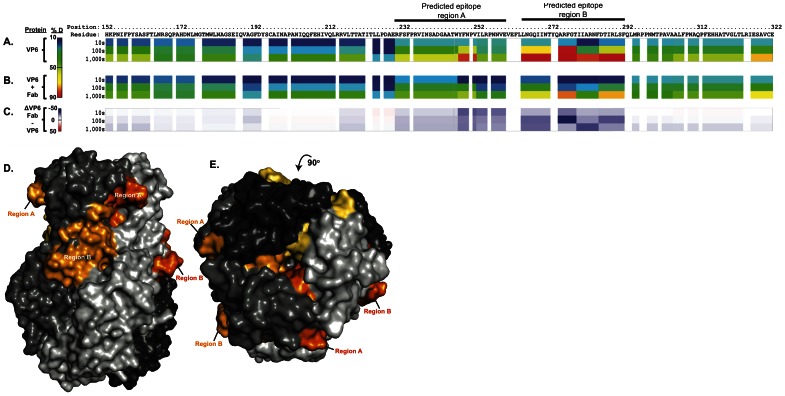
Determination of VP6 epitope for RV6-26 by deuterium exchange mass spectroscopy. Ribbon map showing percent deuterated (% D) of VP6 alone (A), or VP6 bound to RV6-26 Fab (B). The top row shows the residue position number, the second row shows the residue and the rest of the rows show protein dynamic features at different on-exchange time points (10, 100, or 1,000 seconds [s]). As indicated in the colored bar, cold colors suggest relatively stable regions and warm colors suggest relatively flexible regions. All prolines are shown in white because prolines do not have amide hydrogens. Residues uncovered by surface deuteration are also shown in white. (C) Difference map showing the influence of RV6-26 Fab binding to VP6 indicated by changes in % D. Blue suggests the regions that exchange slower upon Fab binding; red suggests the regions that exchange faster upon Fab binding. (D) Side view of the predicted epitope regions of RV6-26 Fab on the VP6 structure (PDB-ID 1QHD). The different shades of gray represent the three protomers that make up the VP6 trimer, and the different shades of orange represent the predicted epitope regions A and B mapped on each protomer. Region A on one protomer and region B on a different protomer are visible. (E) The top view of the VP6 trimer with all the predicted epitope regions visible on the structure.

Next we sought to determine fine details of the epitope recognized by RV6-26. The contact surfaces of the Fabs in the Type I channels were not present in the cryo-EM reconstructions, because the symmetrical distribution resulted in averaging out of the signal at those surfaces. Therefore, we determined the epitope using a different technique, enhanced amide hydrogen/deuterium exchange mass spectroscopy (DXMS). The principle of DXMS analysis is that amide hydrogens on surface residues of proteins exchange readily, but exchange of particular residues can be blocked by antibody binding, suggesting the residues comprising the epitope. Using DXMS, we identified the epitope of RV6-26 on VP6, which involved peptides derived from regions including residues 231–260 and 265–292 (Figure 4A). When identified on a space-filling model of the atomic resolution structure of the VP6 trimer, it became apparent that two separate regions (which we designated region A [amino acids 231–260] or B [amino acids 265–292]) on each VP6 protomer were recognized by the Fab. This epitope region is within a β-sheet that lies on the exposed side of VP6, part of which is exposed inside the transcriptional pore. It should be noted only one protomer of the VP6 trimer presents these surfaces to the inside of the five-fold channel. When we examined the arrangement of the epitope regions A and B from each protomer in the configuration of VP6 trimers on the DLPs, it was clear that the Fab could only bind to regions A and B when they formed a continuous epitope at the junction of two protomers (Figure 4D & E); an Fab could not bind to region A and B on a single protomer. These data indicate that RV6-26 recognizes a quaternary epitope that is formed as a continuous surface using regions from two protomers of the same trimer.

### RV6-26 covers an important area of electrostatic charge on VP6 that may be critical for mRNA transport

**Figure 5:**
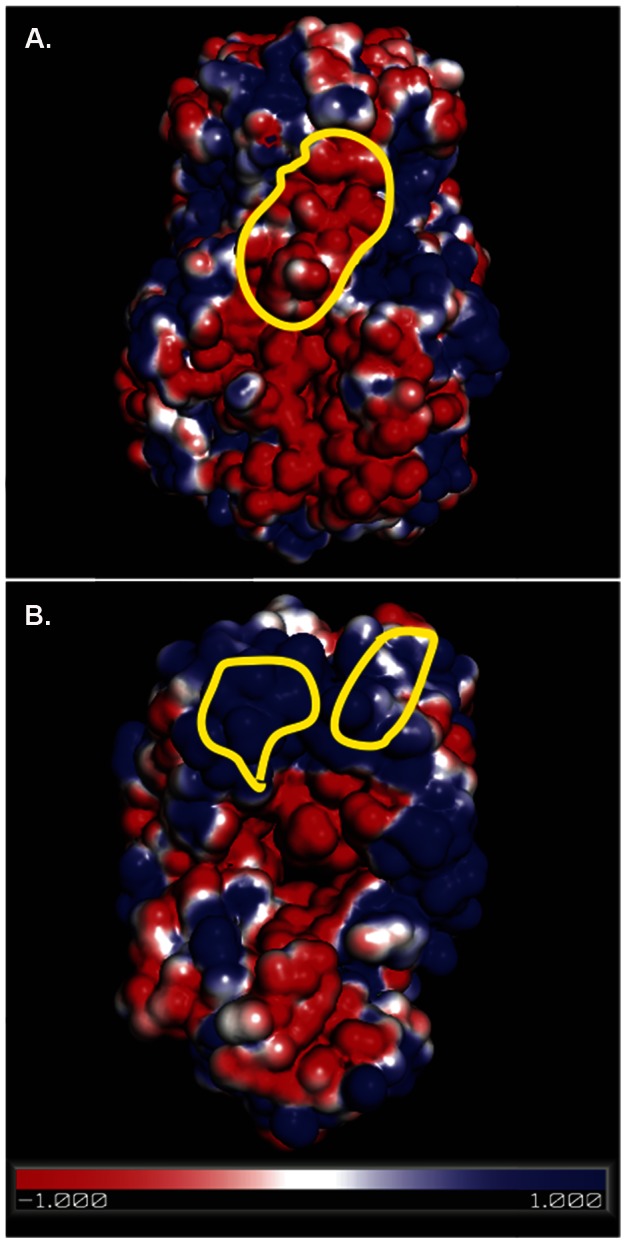
Electrostatic analysis of the binding surfaces involved in interaction between RV6-26 Fab and VP6. The surface electrostatic potential of the VP6 trimer (A), lateral view, or the RV6-26 Fab (B), with red or blue for negative or positive charges, respectively. The yellow circles indicate region B of the VP6 epitope (A) and the heavy and light chain elements of the paratope on Fab RV6-26 (B), as defined by DXMS analysis. See also Figure S2 in File S1 for RV6-26 paratope, as determined by DXMS.

The DXMS-identified epitope recognized by RV6-26 includes a patch of negatively-charged surface at the bottom of the trimer, close to the VP2 layer, inside which transcription occurs (Figure 5). Atomic resolution structures from similar bluetongue virus studies suggested a role for the surface charge of the transcriptional pore, where positively-charged residues in the core layer are positioned to attract negatively-charged nascent mRNA to the mouth of the pore for extrusion [32]. The electrostatic repulsion between the net negative charge of the five-fold channel surface of RV and the viral mRNA is thought to facilitate egress of the mRNA transcript by increasing its fluidity [33]. It is apparent that binding of RV6-26 to its epitope likely would inhibit RV transcription [33]. Binding of RV6-26 to its epitope could partially inhibit RV transcription by covering one of the five negatively-charged patches within the Type I channel. It is of interest that this negatively-charged patch of residues in the channel is highly conserved among diverse rotavirus strains (Figure S1 and Table S1 in File S1).

### Identification of the Fab paratope using DXMS

The residues on the antibody surface that interacted with VP6 (paratope) also were determined by DXMS. Residues 52–59, corresponding to the hypervariable complementarity-determining region 2 of the heavy chain (HCDR2) was shown to be strongly protected from exchange upon complex formation. In addition, residues 67–89 and 95–113 were implicated to be involved in the antigen-antibody interaction. The light chain residues 25–42 and 85–94 also were shown to be protected strongly upon complex formation. Poisson-Boltzmann analysis of the Fab electrostatic surface show that both paratope regions in the heavy and light chains resided in positively-charged regions of the Fab surface (Figure 5B; paratope shown in Figure S2 in File S1).

### Fine details of the Fab-VP6 interaction

**Figure 6:**
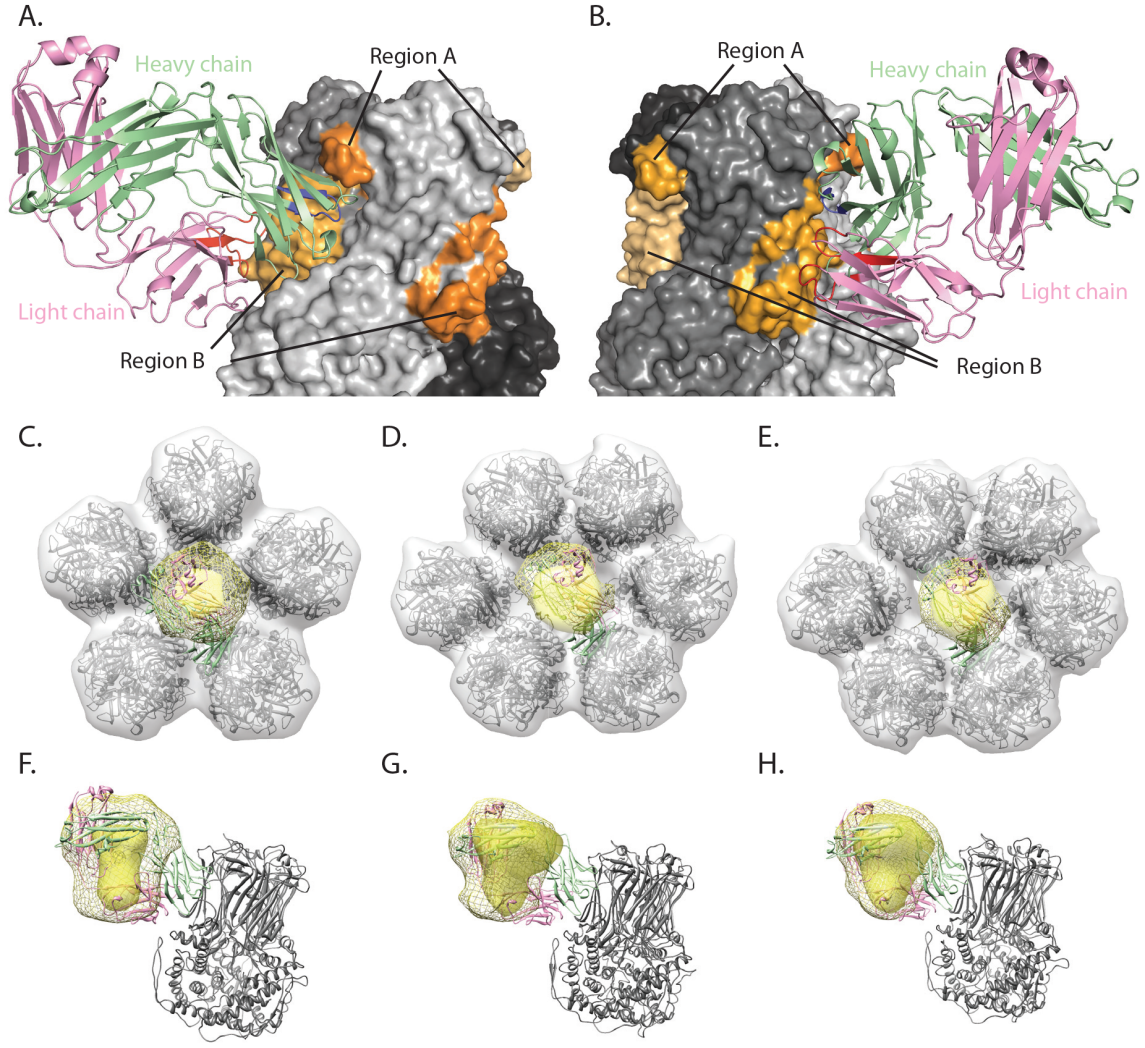
Computer-generated model of VP6-RV6-26 conformation and comparison to predicted epitope regions and cryo-EM density maps. The model was generated with RosettaDock, using DXMS-predicted epitope and paratope regions as restraints during docking. (A) RV6-26 bound to VP6 at a quaternary epitope made up of region A on one VP6 protomer and region B of a second VP6 protomer. Shades of gray represent the three protomers that make up the VP6 trimer, and the shades of orange represent the DXMS-predicted epitope regions mapped on each protomer. Heavy and light chains are green and violet, respectively. DXMS-predicted paratope regions for heavy and light chain are colored blue and red, respectively. (B) Horizontal rotation of frame A. (C–E) Fit of representative model into experimental and simulated cryo-EM density of Type I, II, & III channels, respectively. Density attributed to Fab is yellow with simulated Fab density shown as mesh. Experimental VP6 density is gray (simulated density not shown). Cryo-EM density is overlaid on VP6 crystal structure (PDB-ID 3KZ4). Fab interacting with a single VP6 trimer is displayed as cartoon with light chain colored pink and heavy chain colored green. (F–H) Side view of experimental and simulated Fab density for Type I, II, and III channels, respectively, with coloring as in frame C–E.

We used computational docking with Rosetta to determine the fine specificity of the interaction. We docked atomic resolution structures of the RV6-26 Fab (PDB-ID 4HFW) and the VP6 trimer (PDB-ID 1QHD), both determined by crystallography, using the DXMS-determined epitope and paratope as restraints to guide the docking. Docking calculations were seeded from manually placed antibody locations that matched the DXMS and cryo-EM experimental data and from previously generated models [34]. Trajectories with good predicted binding energies converged on a similar overall conformation of the RV6-26 variable domain region relative to the VP6 trimer, *i.e.*, a single low-energy binding mode consistent with the experimental DXMS data was identified. The interaction confirmed the quaternary nature of the epitope, with the antibody interacting with both region A and region B of adjacent VP6 protomers within a single VP6 trimer (Figure 6A–B). Quaternary interaction was mediated largely by the heavy chain of RV6-26, which interacts with region A and region B, while the light chain predominantly interacted with region B. Significant contact was generated by HCDR2, in agreement with previous studies [26], [34]. To test agreement with the cryo-EM reconstruction, we simulated densities of models from this binding mode assuming equal occupancy of each Fab-trimer interface in the Type I, II, and III channels (Figure 6C–H). There are five epitopes at the Type I channel, but modeling indicates that steric hindrance between Fab fragments limits the number of Fab fragments bound per channel to a maximum of one. Similarly at the Type II and III channels, although there are six epitopes per channel there is only space for one Fab fragment to bind per channel. Significant correlation was observed between the models with the described conformation and the experimental density, with cross-correlation coefficients of 0.51, 0.72, or 0.77 for Type I, II, or III channels, respectively.

Correlation of simulated and experimental cryo-EM densities was highly dependent on even small changes (changes that did not alter the Fab-trimer orientation) in the interaction angle between the Fab and VP6 trimer. We selected the model with highest cross-correlation coefficient values for further analysis, and conclude that the cryo-EM and DXMS datasets are complementary, contributing different and important information to the model. We hypothesize that formation of the complex alters the elbow angle [35] of RV6-26 relative to the angle observed in the crystal structure of the Fab alone. These results corroborate the experimental findings, providing a general mechanism of interaction between RV6-26 and VP6.

### Fitting of cryo-EM data for Fab-DLP complexes to atomic models for DLP or TLP

**Figure 7:**
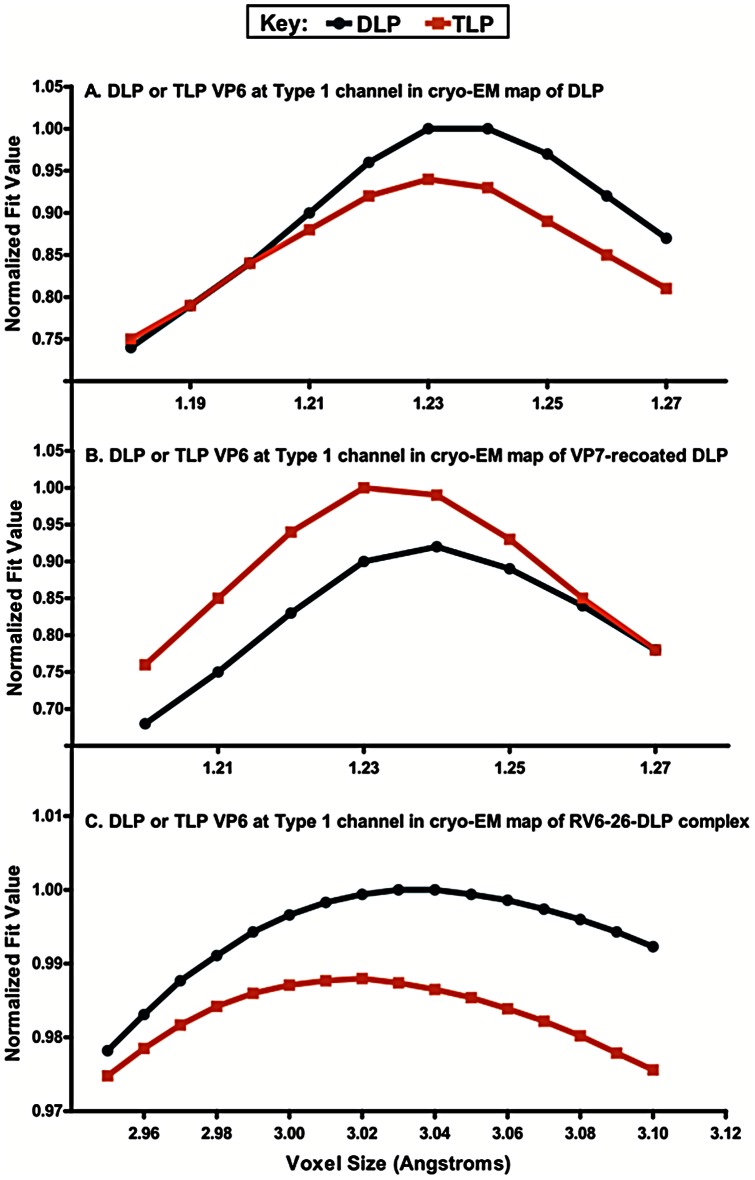
Docking of coordinates for a ring of five VP6 trimers at the Type I channel into cryoEM maps of DLP, VP7 recoated DLP, and the RV6-26-DLP complex. (A) Coordinates for a ring of five VP6 trimers, extracted from the crystal structures of the rotavirus DLP (PDB-ID 3KZ4, blue) and the infectious rotavirus particle (PDB-ID 3N09, orange), docked into a segment of the DLP cryoEM structure (EM Data Bank EMD-1460). (B) Same coordinates docked into a segment of the VP7 recoated DLP cryoEM structure (EM Data Bank EMD-1571). (C) Same coordinates docked into a segment of the RV6-26-DLP complex cryoEM structure. The voxel sizes of the cryoEM density maps (1.21 Å/voxel for DLP and VP7 recoated DLP; 3.02 Å/voxel) were varied plus or minus a few percent to find maximum fit values. Fit values were reported by the UCSF Chimera Fit-In-Map function (Chimera, version 1.5.3) and are shown normalized in each panel.

We next investigated the structural impact of binding of mAb 6-26 to VP6 in the Type I channels. We considered two possibilities regarding the structural effects. One model was that the antibody sterically blocks the channel through which RNAs traffic. The other possibility was that binding of RV6-26 Fab to DLP might induce a conformational change to the global structure of the VP6 layer, such that the bound structure resembled the change this layer undergoes upon VP7 binding during rotavirus assembly, specifically at the five-fold symmetrical transcriptional pore [13]. To investigate these possibilities, we fitted atomic models derived from a crystallographic study of DLP (PDB-ID 3KZ4) or a cryo-EM study of TLP (infectious rotavirus particle; PDB-ID 3N09; [13]) into the cryo-EM density of the RV6-26-DLP complex. These two coordinate sets differ in that the TLP coordinates display a tighter ring of 5 VP6 trimers as well as a narrower Type 1 channel within the ring of five VP6 trimers at the 5-fold axis. The assessment of the RV6-26-DLP conformation involved fitting coordinates for the ring of five VP6 trimers from the DLP or TLP to the corresponding RV6-26-DLP cryo-EM density segment with the Fit-In-Map feature of Chimera software. The idea behind this test was that it would be easier to distinguish between the fit of the two types of VP6 trimer rings than it would be to accurately measure the diameter of the transcriptional pore given the resolution of the RV6-26-DLP cryo-EM structure (10.9 Å). To validate the accuracy of the fitting function for comparison of RV structures, we first fitted either the DLP or TLP coordinate sets into a density segment from a cryoEM structure of DLP (Figure 7A) or a cryoEM structure of a VP7 recoated DLP (Figure 7B). As expected, a higher fitting correlation was observed with DLP coordinates into the DLP density map and with TLP coordinates into the VP7 recoated DLP density map. Next we determined whether the arrangement of VP6 molecules at the Type I channel within the RV6-26 Fab-DLP complex most resembled DLP or the conformationally-altered arrangement found in TLPs. We found a higher fitting correlation of the DLP coordinate set to the RV6-26-DLP complex (Figure 7C). This result indicates that binding of the RV6-26 Fab does not induce a conformational change in the ring of five VP6 trimers at the five-fold axis of the DLP. Therefore, the data suggest a mechanism of inhibition of the transcriptional pore is steric hindrance of the pore, rather than induced conformational change.

## Discussion

These studies are of interest as the data represent the first example of which we are aware of intracellular neutralization of any pathogen by a human antibody or by an antibody induced by natural infection. The phenomenon of intracellular neutralization has been suggested by studies with murine antibodies to influenza, Sendai virus, and rotavirus induced by experimental infection in model systems, but not previously by any *naturally-occurring* antibody nor by any *human* antibody. The work also attributes for the first time a functional protective role to the dominant (V_H_1-46 gene-encoded) B cell response in humans, which has been shown in extensive previous studies to be directed to VP6 [19]–[22], [26]. Dozens if not hundreds of human clinical surveillance and vaccine studies have failed to make a direct association with the VP6 response and an antiviral effect with mechanistic insight. The reason for this lack of finding of neutralizing activity of human VP6 antibodies is that there has not been a way in previous studies to test the effect of human VP6 IgA antibodies in *intracellular* neutralization. The data here show that the most common antibodies people make following RV infection, which are V_H_1-46 gene-encoded VP6-specific antibodies, are not irrelevant to protection as was previously thought, but rather they have the potential to neutralize virus inside cells when present as IgAs. No such function has ever been attributed in the past to this dominant component of the human B cell response following infection.

Despite the availability of several licensed RV vaccines, the correlates of protective RV immunity have not yet been completely elucidated. The high titers of antibodies directed against rotavirus VP6 protein suggested an important role for anti-VP6 antibodies in RV immunity. Indeed, murine studies have demonstrated that RV VP6 can elicit a protective immune response [36]–[38]. Here, we show that an IgA version of the human antibody RV6-26, encoded by the V_H_1-46 germline gene segment that dominates in the human response to RV, can bind and neutralize RV-DLP intracellularly by inhibiting viral transcription. These studies suggest that intracellular neutralization by naturally-occurring RV VP6-specific IgAs likely contributes significantly to human immunity to RV. It is difficult to define from *in vitro* studies exactly how well the quantitative aspects of these studies correlate with the potency of intracellular neutralization in the human intestine, since the efficiency of both virus replication and antibody transcytosis and inhibitory activity likely differ in polarized cells in the human gut compared to those in our *in vitro* cell line studies. Nevertheless, the studies presented show that intracellular neutralization likely is a significant component of human immunity to RV.

Structural studies using single particle cryo-EM provided insights into the mechanism of inhibition of RV transcription by RV6-26. A previously published high-resolution cryo-EM study of uncoating and recoating of the outer VP7 layer showed a distinct shift in the orientation of the VP6 trimers around the five-fold axes that resulted in a 5 Å decrease in diameter of transcription pore [13]. It was suggested that the uncoating of the VP7 layer in the transcriptionally-incompetent RV particle, and ensuing reorientation of the five-fold VP6 trimers, could serve as a trigger for the activation of RNA synthesis. Previous cryo-EM studies of murine inhibiting or non-inhibiting Fabs postulated either prevention [39] or induction [40] of a conformational change in the VP6 trimers at the transcriptional pore in the inhibiting Fab-complex structures in a similar way as that mediated by VP7. One of the studies suggested no effect of inhibitory Fab binding on DLP conformation [39]. The cryo-EM structures obtained in those Fab studies, however, likely were not high enough resolution to definitively resolve whether or not a conformational change resulted from binding.

In the present study, we did not observe any conformational changes to the five-fold VP6 trimers as a result of RV6-26 binding that resembled those induced by VP7 recoating. Our DLP-RV6-26 complex structure showed that the Fab bound to the VP6 trimers with Fab projecting into the center of the Type I channel (transcriptional pore), suggesting a physical blockade of the pore as the principal mode of inhibition. We determined the epitope of the RV6-26 Fab by DXMS and computational docking using Rosetta, which revealed a quaternary epitope involving an inserted β-hairpin of one VP6 protomer and some pore-exposed residues within a β-sheet of the adjacent VP6 protomer comprising residues 231–260 and 265–292, respectively. The data here indicating the quaternary nature of epitopes recognized by inhibitory human Fabs is consistent with some previous epitope mapping studies of murine inhibitory Fabs. The main difference observed between the inhibiting and non-inhibiting Fab was in the location of their epitopes [39]. The epitope of inhibitory Fabs involved multiple loops on two VP6 protomers, while only one VP6 protomer was involved in binding of the non-inhibitory Fabs. The light chain inserted deeper into the transcription pore, while the heavy chain bound to a region higher up in the pore. Our previous mutagenesis work showed that the HCDR2 loop of RV6-26 is associated with much of the antibody inhibitory function [26]. The structural data presented here may explain in part the dominance of the V_H_1-46 gene segment in the human response to VP6, since the HCDR2 region is encoded by the V_H_ gene segment and is an important structural feature mediating the interaction with the upper domain of the quaternary epitope.

Electrostatic analysis of the surface of the RV6-26 epitope and paratope revealed that the epitope lies within a patch of negatively-charged surface that extends from the base of VP6 towards the bottom of the transcriptional pore. It has been postulated that this negatively-charged surface within the transcription channel may be important for repulsion of negatively charged viral mRNA during egress from the particle, as transcription is initiated within the RV-DLP. Successful threading of an initiated mRNA transcript through the pore may be a critical step required for the transition from transcription initiation to elongation, which is essential for RV to produce mature full-length transcripts [40]. Not surprisingly, the paratope surface of RV6-26 is very positively charged, suggesting that the antigen-antibody interaction may be driven by electrostatic interactions between the antibody and VP6. It is possible that RV6-26 binding to VP6 partially disrupts a charged-based mechanism of guidance of the path of mRNA translocation from the particle core, however our studies did not specifically address the mechanism of RNA threading. The size of the Fab in the Type I channel likely is sufficient to sterically block RNA egress from the channel even if the charge determinants are not essential to replication.

These studies also revealed a pattern of epitope recognition that provides insights into the overall viral architecture, and may have broad implications for antibody recognition of epitopes in the context of whole viral particles. RV6-26 exhibited three different binding patterns at the Type I, II and III channels. Interestingly, reconstructed density in the Type III channel was asymmetric, suggesting tighter interactions with VP6 trimers at the P′ and T′ positions. However, we demonstrated that this asymmetry can be explained by the deviation from a perfect hexagonal pore at the quasi-six-fold axes. This observation does not exclude preferential binding or differential accessibility resulting from this deviation from perfect symmetry. However, neither effect is needed to explain our findings at the resolution of the data. In summary, the cryo-EM structure of the RV6-26-DLP complex, combined with the deuterium exchange mass spectroscopy evaluation of the epitope regions and modeling of the docked Fab fragment crystal structure together indicate that the predominant mode of rotavirus inhibition by RV6-26 is steric hindrance of the DLP transcriptional pore.

## Materials and Methods

### Cells

MA-104 Clone 1 cells (ATCC CRL-2378.1) and Madin Darbin canine kidney (MDCK) cells (ATCC CCL-34) were grown in complete medium (CM) consisting of DMEM with 4.5% glucose (Mediatech) supplemented with 10% (v/v) fetal bovine serum (Invitrogen), 0.1 mM MEM non-essential amino acids (Invitrogen), 100 I.U./mL penicillin, 100 µg/mL streptomycin, 2 mM glutamine and 1 mM sodium pyruvate (all from Mediatech). Infection medium (IM), used when cells were inoculated with rotavirus and cultured, contained all of the above supplements except serum, and trypsin-EDTA (Invitrogen) was added to a final concentration of 1 µg/mL. A lineage of Caco-2 cells (a kind gift from Dr. Blaise Corthésy) derived by passage from a commercial cell line (HTB-37, American Type Culture Collection) were grown in CM supplemented with 0.1% transferrin (Sigma). All cell lines were cultured at 37°C with 5% CO_2_. 293-F cells were grown in FreeStyle 293 serum-free expression medium (Invitrogen). 293-6E cells licensed from the National Research Council of Canada [41], were grown in F17 expression medium (Invitrogen). Culture of 293-F and 293-6E cells was done in shaker flasks at 125 rpm and 8% CO_2_.

### Expression and purification of recombinant antibodies

The isolation of the genes encoding the human VP6-specific monoclonal antibody RV6-26 from a single B cell was described previously [19], and the antibody gene sequences are available in GenBank, accession numbers AF452996 and AF453157. The RV6-26 heavy and light chain variable region genes were sequence-optimized and synthesized as cDNAs (GeneArt). These synthetic genes were cloned into the pEE6.4 expression vector (Lonza) in-frame with a mouse kappa chain leader sequence at the 5′ end and optimized C_L_ and C_H_1 constant domain sequences at the 3′ end, to encode a fully human Fab antibody fragment without affinity tags. The separate plasmids encoding heavy or light chain genes each were transformed into DH5α strain *E. coli* cells for large-scale plasmid DNA preparation (PureYield; Promega). Heavy- and light-chain encoding plasmid DNAs were co-transfected transiently into a high-producing clonal variant of the HEK-293 cell line cells (FreeStyle 293-F cells; Invitrogen) using Polyfect reagent (Qiagen), and the cells were incubated in humidified air with CO_2_ in shaker flasks for 7 days. The supernatant was collected on day 7 and purified by fast protein liquid chromatography using an ÄKTA FPLC device and HiTrap KappaSelect column (GE Healthcare) in D-PBS, and then concentrated with 30 mL Amicon Ultra centrifugal filter units with 30 kDa molecular weight cut-off (Millipore). For higher expression of antibodies as Fabs in some cases, the heavy and light chain variable region cDNAs were cloned (using *Sal*I/*Not*I for light chain and *Eco*RI/*Hin*dIII for heavy chain) into plasmid vectors optimized for high-level protein expression in 293-6E cells and containing C_K_ and C_H_1, for heavy and light chains, respectively [41].

For expression as IgG or IgA, the light chain variable domains were cloned as *Bgl*II/*Not*I fragments into the pLC-huCK plasmid vector and the heavy chain variable domain cDNAs were cloned as *Eco*RI/*Hin*dIII fragments into pHC-huCG1 or pHC-huCA1 plasmid vectors for γ or α chains, respectively [42]. The vectors were kindly provided by Dr. Gary McLean. Equal amounts of light and heavy chain vector DNA were mixed in OptiMEM I (Invitrogen) and complexed with either 25 kDa linear PEI (PolySciences Inc.) or PolyFect (Qiagen) for 10 min (DNA∶transfection reagent = 1∶1.5). For expression of IgA antibodies, pCH-J vector DNA (coding for J chain necessary for dimerization of IgA, a kind gift from Dr. F.E. Johansen) also was added. 293-F or 293-6E cells were transfected transiently with DNA complexes and cultured for 4–6 days. The secreted antibodies were harvested from the culture medium by affinity chromatography using KappaSelect (for Fabs), Protein G (for IgG) or anti-human α chain agarose (for IgA). Assembly of dimeric IgA was confirmed by resolving purified IgA on a Superdex 200 10/300 GL column (GE Healthcare) calibrated with reference proteins of 440, 158, 75 and 44 kDa molecular weight according to the manufacturer's instructions. Recognition of antigen by different molecular forms of the antibodies was determined in an ELISA. Purified RRV DLP were coated in wells of an ELISA plate and blocked with 5% skim milk/PBS-T. Several concentrations of antibodies, designed to normalize to the number of antigen binding sites (Fab = 1; IgG = 2 or IgA = 4) were added. After washing with PBS-T, the bound antibodies were detected with peroxidase-conjugated anti-human κ chain antibodies (Southern Biotech). Color development was performed using Ultra TMB substrate (Pierce), and then stopped with 1 M HCl and optical density determined at 450 nm with a plate reader.

### Crystallization

H5.3 Fab crystals were grown by vapor diffusion of 20 mg/mL protein against a reservoir of 2.0 M ammonium sulfate and 5% vol/vol 2-propanol. Crystals were cryoprotected by brief soaks in mother liquor supplemented with 20% glycerol and cooled in liquid nitrogen.

### Data collection and structure determination

Diffraction data were collected from single crystals at 100 K at sector LS-CAT 21-ID-D at the Advance Photon Source (Argonne, IL). Data were indexed, integrated and scaled with XDS, xdia2, and Scala [43]–[47]. Data collection statistics are given in Table S2 in File S1. 5% of the data were selected randomly as an R_free_ set [48]. Molecular replacement was performed with Molrep [49] by iteratively searching a library of ∼250 Fab fragments. The asymmetric unit contains two Fabs, and both molecules were readily found by molecular replacement. The best solution, obtained with Fab fragment from PDB-ID 1FDL, was identified based on the Contrast score. Side chains of this oriented model that differed from the 626 sequence were trimmed to alanine, and the resulting oriented model was refined in Phenix [50]. Refinement included rigid body refinement of the individual domains, simulated annealing, positional, individual B-factor, and TLS. Loops were rebuilt and side chains added in COOT [51] using simulated annealing composite omit maps [52] generated by Phenix [50]. Non-crystallographic restraints were imposed throughout refinement. TLS refinement [53] was incorporated in the final rounds of refinement using TLS groups identified using Phenix. The refined model consists two molecules, molecule A contains light chain amino acids 1–212 and heavy chain amino acids 1–220 molecule be contains light chain residues 2–211 and heavy chain residues 1–221. In addition, two bound sulfates and 335 waters were included in the final structure. The final R_factor_ was 20.07%, the R_free_ is 23.50 for data between 50 and 2.6 Å. Additional data and model statistics are given in Table S2 in File S1. The structure has been deposited in the Protein Data Bank under accession code 4HFW.

### Preparation and purification of rotavirus double-layered virus particles (DLPs)

A strain of rhesus rotavirus (RRV) was kindly provided by Susana López (Universidad Nacional Autónoma de México). Virus was inoculated onto cell culture monolayers of MA-104 cells (CRL-2378.1; ATCC, Manassas, VA) at low MOI. When cell monolayers exhibited significant cytopathic effect, the supernatant and cell fractions were collected and virus was isolated by ultracentrifugation in a Sorvall Discovery 90SE centrifuge with a Surespin 630 rotor at 20,000 rpm for 1.5 hours at 4°C. The resulting pellet was resuspended in Earle's Balanced Salt Solution (GIBCO) and the cellular debris was removed by addition of 1,1,2-trichloro-trifluoroethane (EMD Chemicals) and blending. The suspension then was centrifuged at 2,000 rpm for 0.5 hours at 4°C to separate virus from cell debris. Virus particles then were collected and purified by ultracentrifugation through a CsCl cushion at 20,000 rpm for 1.5 hours. To prepare concentrated DLPs, the collected viral particles were treated with 10 mM EGTA (Lonza) for 5 minutes at room temperature to remove the outer VP4 and VP7 protein layers of the rotavirus triple-layered particles. The resulting DLPs were purified further by CsCl density gradient ultracentrifugation at 29,700 rpm for 20 hours. The visible band containing DLPs was collected and concentrated further by ultracentrifugation in the same CsCl density gradient at 29,700 rpm for 2 hours. The band then was collected and dialyzed into Tris-buffered saline (Mediatech).

### Cryo-electron microscopy

Purified DLPs were mixed with five-fold molar excess of purified RV6-26 Fab protein, and then the excess antibody was removed using centrifugal filters with Sephadex G-50 resin. The complexes of RV DLPs with RV6-26 Fab in Tris-buffered saline pH 8.0 with 0.5% glycerol were applied to freshly prepared electron microscopy (EM) grids with a holey carbon film. The excess liquid from a 2–3 µL droplet was blotted away with filter paper. Another 2–3 µL droplet was added to the same EM grid and excess liquid was blotted as before. The sample grid was plunged immediately into liquid ethane cooled by liquid nitrogen. All data was collected on an FEI Tecnai Polara microscope equipped with a field emission gun (FEG) operating at 300 kV in nanoprobe mode. The sample grids were maintained at liquid nitrogen temperature during data acquisition. Images were recorded digitally on a Gatan UltraScan 4000 (4 k×4 k) CCD camera. Micrographs were collected with a defocus range of 0.5–5 µm and with an absolute magnification of 199,000×.

### Image processing and reconstruction

Individual particle images were centered manually, cropped and binned using in-house scripts in conjunction with IMAGIC and EMAN image processing suites. A total of 3,029 particle images was picked and processed in this dataset. Images were binned and stacked with 6.0 and 3.0 Å pixel sizes. 6.0 Å stacks were used for initial CTF parameter determination using CTFFIND3. Orientation, magnification and defocus parameter determination and refinement were carried out using FREALIGN. Image processing was carried out through 34 rounds of FREALIGN refinement, converging at a resolution of 10.9 Å, at a pixel binning of 3.0 Å, and the data was deposited in EM Data Bank (EMD - *Pending*). Resolution was determined as measured by the Fourier shell correlation (FSC) 0.5 criterion. Icosahedral symmetry was imposed during data processing. Docking of coordinates for a ring of five VP6 trimers extracted from the crystal structures of the rotavirus DLP (PDB-ID 3KZ4) and the infectious rotavirus particle (PDB-ID 3N09) was performed with the UCSF Chimera Fit-In-Map function. The same coordinate sets also docked into segments of the DLP cryoEM structure (EM Data Bank EMD-1460), the VP7 recoated DLP cryoEM structure (EM Data Bank EMD-1571), and the cryo-EM structure of the RV6-26-DLP complex. The voxel size of each cryo-EM density map was varied in Chimera by plus or minus a few percent to maximize the reported average map value. The resulting set of average map values were normalized and plotted for each cryo-EM structure (Figure 7). In addition, we also converted the coordinates for a ring of five VP6 trimers at the 5-fold axis from the DLP and TLP atomic resolution structures into density maps at 10.9 Å resolution to simulate the cryo-EM density. Fitting of these two simulated density maps for a ring of five VP6 trimers into the cryo-EM structure of the RV6-26-DLP complex also indicated a better fit for the DLP structure.

### Inhibition of RV *in vitro* transcription by anti-RV VP6 antibodies

An *in vitro* transcription assay, measuring RNA by quantitative PCR, was used to determine the ability of our anti-rotavirus antibodies to inhibit rotavirus transcription. The *in vitro* transcription assay format was similar to one that was described previously [24]. Briefly, purified RRV-DLPs were activated with 5 mM EDTA at 37°C for 30 minutes. Equal volumes of activated DLPs at 50 µg/mL concentration and purified Fabs in a solution containing 400 nM combining sites were mixed and incubated at 37°C for 1 hour. Rotavirus *in vitro* transcription was performed per manufacturer's instructions using commercial reagents (selected components of the Riboprobe system – SP6 [Promega]), with transcription mediated by the viral RNA-dependent RNA polymerase, not the SP6 DNA-dependent RNA polymerase). The RRV-DLP-Fab mixtures were added to the transcription reaction and incubated at 40°C for 1 hour. Rotavirus cDNA was synthesized from RNA transcripts using GoScript Reverse Transcriptase (Promega) and quantified by quantitative PCR as previously described [54] using the following primers: LoVP6_RT_FWD: GATTCACAAACTGCAGATTCGA, and LoVP6_RT_BCK: AGGTCGCTGGATTCGACTATTC.

DNAs were detected using the Express SYBR GreenER qPCR kit (Invitrogen) and monitored on a Cepheid SmartCycler. Amplification conditions used were: 95°C 15 min; 40 cycles of 95°C–15 s, 62°C–30 s, 72°C–60 s; 72°C–10 min. Melt curve conditions were: 95°C for 5 s followed by change from 65°C to 95°C at a rate of 0.5°C/s. Quantification of viral RNA was done by extrapolating from a standard curve constructed using amplified cDNA from known concentrations purified viral RNA.

### VP6 Fragment Expression and Purification

A DNA copy of the VP6 sequence encoding residues 147–339 was amplified from a full-length VP6 DNA construct by polymerase chain reaction (PCR) with Pfu Ultra polymerase (Stratagene) using the following primers:

Forward: 5′-GGAAGGccatggccCGGACCGGCTTCACCTTCCAC-3′

Reverse: 5′- GTGGTGctcgagGCTGGCGTCGGCCAGCACGC-3′

The PCR product was cloned into the pET28a vector (Novagen) using the NcoI and XhoI restriction sites. For expression and purification, the pET28a vector containing the head domain construct was transformed into BL21(DE3) *E. coli* strain. Cells were grown in 1 L of LB medium to an OD_600_ of 0.6 and protein expression then induced by addition of IPTG to a final concentration of 0.1 mM and allowed to grow overnight at 20°C. Cells were harvested by centrifugation and disrupted with a French pressure cell press in 50 mM sodium phosphate buffer, pH 8.0. The soluble fraction was clarified and applied over a Ni-NTA column. The VP6 protein then was eluted with the above buffer with 400 mM imidazole. The eluate was concentrated in Amicon Ultra filter tubes (Millipore) and passed through a Superdex S200 column for additional purification.

### Deuterium exchange mass spectrometry (DXMS)

DXMS can be used to rapidly map key regions/residues in protein-protein interactions and can define a more extensive interaction domain than point mutants alone. We performed comparative DXMS studies with a VP6 fragment (VP6f) construct alone or with VP6f in complex with RV6-26 Fab. Prior to the deuteration studies, quench conditions optimal for maximum peptide fragmentation were determined, as previously described [55], [56]. Complexes of VP6f and Fab were prepared by mixing VP6f and RV6-26 Fab at 1∶1.4 stoichiometric ratio, and incubating the mixture at 0°C for 30 minutes. The concept was to use an excess amount of Fab in the binding experiment to saturate all of the binding sites on VP6, and then to compare the hydrogen-deuterium exchange profile difference between VP6 alone and VP6-Fab complex. In similar experiments for paratope mapping on the RV6-26 Fab, we used an excess amount of VP6 to form complexes to ensure that all binding sites on Fab were occupied by VP6, and then compared the results between Fab alone and VP6-Fab complex.

Functional hydrogen-deuterium exchange reaction of free VP6f was initiated by diluting 0.7 µL of stock solution into 1.3 µL of H_2_O buffer (8.3 mM Tris, 150 mM NaCl, in H_2_O, pH 7.15), and then mixed with 6 µL of D_2_O buffer (8.3 mM Tris, 150 mM NaCl, in D_2_O, pD_READ_ 7.15). At 10 sec, 100 sec or 1,000 sec, 12 µL of optimized quench reagent (0.8M GuHCl in 0.8% formic acid) was added to the respective samples and then samples were frozen at −80°C. The functionally-deuterated antibody-bound VP6f samples were prepared by diluting 1.5 µL of complex solution (10.4 mg/mL for VP6 - RV6-26) into 0.5 µL of non-deuterated buffer, and then mixed with 6 µL of D_2_O buffer (8.3 mM Tris, 150 mM NaCl, in D_2_O, pD_READ_ 7.15). At 10 sec, 100 sec or 1,000 sec, 12 µL of optimized quench reagent was added to the respective samples and then samples were frozen at −80°C. In addition, non-deuterated samples (incubated in H_2_O buffer mentioned above) and equilibrium-deuterated back-exchange control samples (incubated in D_2_O buffer containing 0.5% formic acid overnight at 25°C) were prepared as previously described [55]–[57]. Later, the samples were thawed automatically on ice and then immediately passed over an AL-20-pepsin column (16 µL bed volume, 30 mg/mL porcine pepsin (Sigma)), which was run at a flow rate of 20 µL/min with 0.05% trifluoroacetic acid. The resulting peptides were collected on a C18 trap and separated using a C18 reversed phase column (Vydac) running a linear gradient of 0.046% (v/v) trifluoroacetic acid, 6.4% (v/v) acetonitrile to 0.03% (v/v) trifluoroacetic acid, 38.4% (v/v) acetonitrile over 30 min with column effluent directed into an LCQ mass spectrometer (Thermo-Finnigan LCQ Classic) for epitope mapping or into an Orbitrap Elite mass spectrometer for paratope mapping. Data were acquired in both data-dependent MS∶MS mode and MS1 profile mode. SEQUEST software (Thermo Finnigan Inc.) was used to identify the sequence of the peptide ions. The centroids of the isotopic envelopes of non-deuterated, functionally-deuterated, or fully-deuterated peptides were measured using DXMS Explorer (Sierra Analytics Inc., Modesto, CA), and then converted to corresponding deuteration levels with corrections for back-exchange [58].

### Transcytosis assays

MDCK cells were grown on Transwell inserts (clear, 0.33 cm^2^ growth area, 0.4 µm pore size; Corning) in complete medium. Formation of a tight, polarized cell monolayer was monitored by measuring trans-epithelial electrical resistance (TEER) using an Endohm cup chamber and EVOM2 resistance meter (WPI Inc.). IgG and IgA antibodies were suspended in the medium in the basal compartment at different concentrations and the medium in the insert was assayed for the transcytosed antibodies in a capture ELISA. Anti-human κ chain antibody was coated in the wells of an ELISA plate and, after blocking with 5% skim milk/PBS-T, the supernatant from the inserts was added to the wells. After washing, the bound antibodies were detected using anti-human IgG (Fc-specific) or anti-human IgA (Fc-specific) peroxidase conjugates (Southern Biotech) for IgG and IgA, respectively.

### Intracellular neutralization assay

Caco-2 cells were grown on Transwell inserts for about 21 days in complete medium with 0.1% transferrin. The medium in the Transwell insert was replaced with medium devoid of serum for 24 h before the inoculation with rotavirus. Antibodies were suspended (20 µg/mL) in the medium in the basal compartment 4–6 h prior to virus inoculation. Virus stocks were activated with 1 µg/mL trypsin for 30 min at 37°C, and cells were inoculated (MOI = 5) for 1 h at ambient temperature. The virus then was replaced with infection medium and culture continued for another 16 h. Virus in the supernatants was titered by inoculating MA104 cell culture monolayers for 1 h at ambient temperature and culturing for another 16 h in infection medium. Cells also were inoculated with serial dilutions of virus stock in order to construct a standard curve. The cells then were washed and treated with cold acetone for fixation and permeabilization. After blocking the wells with 3% BSA/PBS, the virus particles were stained with polyclonal goat anti-rotavirus antibodies followed by anti-goat antibodies labeled with either IRDye 800 CW (Licor) or Alexa 568 (Invitrogen). The amount of virus was quantified by either scanning the plate on an Odyssey scanner or counting the fluorescent foci.

### Computational docking of Fab to VP6 epitope

Prior to docking, VP6 trimer and RV6-26 were relaxed using the Rosetta FastRelax protocol with a harmonic restraint placed on all Cα pairs within 8 Å using a standard deviation of 0.5 Å. The VP6 trimer was submitted to five rounds of relax, while the antibody variable domain underwent rounds of relaxation until energy and geometry converged. The ten top-scoring VP6 and RV6-26 models were carried into docking, with each docking run performed with all pairwise combinations of antibody and antigen. To decrease the time required for generation of each model, docking was performed with a single VP6 trimer and only the variable domains of the antibody. We performed a conformational search during docking, allowing 3 Å translational and 8° rotational perturbations of the antibody relative to antigen during the low-resolution search and 0.1 Å translational and 3° rotational perturbations during the high resolution search [13]. Several different approaches for incorporation of DXMS data were explored. First, interaction between surface residues within the inferred epitope and paratope was encouraged through addition of distance restraints to C-α atoms across the interface. Alternatively, non-overlapping segments of the epitope and paratope were determined from the DXMS data, and each segment formed the basis for a single restraint. In each case, the restraint was fulfilled if any amino acid C-α atom within a contiguous peptide segment had a Euclidean distance below 8 Å to a C-α atom of the partner protein. A flat harmonic penalty with a standard deviation of 2 Å was added to the score if no amino acid within the segment fulfilled the restraint. All Rosetta experiments were performed within the RosettaScripts framework [35] with Rosetta revision 49262. To simulate cryo-EM densities, a fully occupied channel was generated through alignment of the VP6:RV6-26 complex model to each trimer in a channel extracted from the crystal structure of the rotavirus DLP. Antibody constant domains were added back through alignment of the RV6-26 Fab crystal structure with the model. Cryo-EM densities of the fully saturated channel were simulated with BCL::PDBToDensity [40] at a resolution of 10.9 Å and voxel size of 3.02 Å^3^ and Gaussian noise added to the simulated map for an overall CCC of 0.5 for the simulated map to the noised map. Cross-correlation coefficients of simulated and experimental antibody densities were calculated within Chimera after optimally aligning the VP6 channel.

## Supporting Information

Figure S1 **Alignment of group A and non-group A rotavirus VP6 sequences showing conservation of the negatively-charged surface electrostatic potential in the VP6 trimer.** Alignment of representative sequences from group A and non-group A rotavirus VP6 sequences (specific strains are shown in table below) was performed using ClustalW2, the multiple sequence alignment computer program. Numbers at the top indicate the residue number. Small letters represent 1-letter residue abbreviations of the consensus VP6 sequence based on the aligned representative VP6 sequences. The gray bar graphs represent identity histograms showing conservation levels of individual residues at each position. The dashes within the sequences indicate absence of residues from the specific strain sequences accessed from GenBank. Residues below the solid black line indicate those that are shown to contribute to the negatively-charged electrostatic patch within the transcriptional pore formed by VP6 trimers.
(PDF)

Table S1 **Table S1, Sources of the VP6 sequences used for alignment analysis of conserved negative patch. Table S2, Data collection and refinement statistics.**
(PDF)

Figure S2 **RV6-26 Fab paratope as determined by deuterium exchange mass spectroscopy.** (A) Side view of the predicted paratope regions of RV6-26 Fab on the antibody structure (PDB-ID 4HFW). The color scheme is as used in Figure 6. The antibody light chain is represented in pink and the heavy chain is shown in pale green. Red and blue depict the antibody regions predicted to form the RV6-26 Fab paratope in the light and heavy chains respectively. (B) The top view of the RV6-26 Fab showing the antigen combining region with the DXMS-predicted regions shown to be involved in antigen interactions represented in red and blue on the light and heavy chains, respectively.
(PDF)

Table S2 **Data collection and refinement statistics.**
(PDF)

## References

[1] Estes MK (2001) Rotavirus and their replication. In: Knipe DM, Howley PM, editors. Fields Virology. Philadelphia, PA: Lippincott Williams and Wilkins. pp. 1747–1785.

[2] Kapikian A (2001) A rotavirus vaccine for prevention of severe diarrhoea of infants and young children: development, utilization and withdrawal. Novartis Found Symp 238: 153–171 (Discussion, 238:171–179.). 11444025 10.1002/0470846534.ch10

[3] Yeager M, Dryden KA, Olson NH, Greenberg HB, Baker TS (1990) Three-dimensional structure of rhesus rotavirus by cryoelectron microscopy and image reconstruction. J Cell Biol 110: 2133–2144. 2161857 10.1083/jcb.110.6.2133 PMC2116141

[4] Prasad BVV, Rothnagel R, Zeng CQY, Jakana J, Lawton JA, et al (1996) Visualization of ordered genomic RNA and localization of transcriptional complexes in rotavirus. Nature 382: 471–473. 8684490 10.1038/382471a0

[5] Estes MK, Kapikian AZ (2007) Rotaviruses. In: Knipe DM, Howley PM, editors. Fields Virology. Philadelphia, PA: Lippincott Williams and Wilkins. pp. 1918–1974.

[6] Prasad BV, Wang GJ, Clerx JP, Chiu W (1988) Three-dimensional structure of rotavirus. J Mol Biol 199: 269–275. 2832610 10.1016/0022-2836(88)90313-0

[7] Zhang X, Settembre E, Xu C, Dormitzer PR, Bellamy R, et al (2008) Near-atomic resolution using electron cryomicroscopy and single-particle reconstruction. Proceedings of the National Academy of Sciences of the United States of America 105: 1867–1872. 18238898 10.1073/pnas.0711623105 PMC2542862

[8] Ludert JE, Gil F, Liprandi F, Esparza J (1986) The structure of the rotavirus inner capsid studied by electron microscopy of chemically disrupted particles. J Gen Virol 67 (Pt 8) 1721–1725. 3016160 10.1099/0022-1317-67-8-1721

[9] Roseto A, Escaig J, Delain E, Cohen J, Scherrer R (1979) Structure of rotaviruses as studied by the freeze-drying technique. Virology 98: 471–475. 228484 10.1016/0042-6822(79)90571-3

[10] Shaw AL, Rothnagel R, Chen D, Ramig RF, Chiu W, et al (1993) Three-dimensional visualization of the rotavirus hemagglutinin structure. Cell 74: 693–701. 8395350 10.1016/0092-8674(93)90516-S PMC7133302

[11] Yeager M, Berriman JA, Baker TS, Bellamy AR (1994) Three-dimensional structure of the rotavirus haemagglutinin VP4 by cryo-electron microscopy and difference map analysis. EMBO J 13: 1011–1018. 8131735 10.1002/j.1460-2075.1994.tb06349.x PMC394908

[12] Aoki ST, Settembre EC, Trask SD, Greenberg HB, Harrison SC, et al (2009) Structure of rotavirus outer-layer protein VP7 bound with a neutralizing Fab. Science 324: 1444–1447. 19520960 10.1126/science.1170481 PMC2995306

[13] Chen JZ, Settembre EC, Aoki ST, Zhang X, Bellamy AR, et al (2009) Molecular interactions in rotavirus assembly and uncoating seen by high-resolution cryo-EM. Proceedings of the National Academy of Sciences of the United States of America 106: 10644–10648. 19487668 10.1073/pnas.0904024106 PMC2689313

[14] Cohen J, Laporte J, Charpilienne A, Scherrer R (1979) Activation of rotavirus RNA polymerase by calcium chelation. Archives of Virology 60: 177–186. 41504 10.1007/BF01317489

[15] Lawton JA, Estes MK, Prasad BV (1997) Three-dimensional visualization of mRNA release from actively transcribing rotavirus particles. Nat Struct Biol 4: 118–121. 9033591 10.1038/nsb0297-118

[16] Libersou S, Siebert X, Ouldali M, Estrozi LF, Navaza J, et al (2008) Geometric mismatches within the concentric layers of rotavirus particles: a potential regulatory switch of viral particle transcription activity. Journal of Virology 82: 2844–2852. 18184711 10.1128/JVI.02268-07 PMC2258973

[17] Offit PA, Blavat G (1986) Identification of the two rotavirus genes determining neutralization specificities. J Virol 57: 376–378. 3001359 10.1128/jvi.57.1.376-378.1986 PMC252740

[18] Offit PA, Blavat G, Greenberg HB, Clark HF (1986) Molecular basis of rotavirus virulence: role of gene segment 4. J Virol 57: 46–49. 3001364 10.1128/jvi.57.1.46-49.1986 PMC252697

[19] Weitkamp JH, Kallewaard N, Kusuhara K, Bures E, Williams JV, et al (2003) Infant and adult human B cell responses to rotavirus share common immunodominant variable gene repertoires. J Immunol 171: 4680–4688. 14568943 10.4049/jimmunol.171.9.4680

[20] Weitkamp JH, Kallewaard NL, Bowen AL, Lafleur BJ, Greenberg HB, et al (2005) VH1-46 is the dominant immunoglobulin heavy chain gene segment in rotavirus-specific memory B cells expressing the intestinal homing receptor alpha4beta7. J Immunol 174: 3454–3460. 15749880 10.4049/jimmunol.174.6.3454

[21] Weitkamp JH, Lafleur BJ, Crowe JE Jr (2006) Rotavirus-specific CD5+ B cells in young children exhibit a distinct antibody repertoire compared with CD5- B cells. Hum Immunol 67: 33–42. 16698423 10.1016/j.humimm.2006.02.024

[22] Weitkamp JH, Lafleur BJ, Greenberg HB, Crowe JE Jr (2005) Natural evolution of a human virus-specific antibody gene repertoire by somatic hypermutation requires both hotspot-directed and randomly-directed processes. Hum Immunol 66: 666–676. 15993712 10.1016/j.humimm.2005.02.008

[23] Burns JW, Siadat-Pajouh M, Krishnaney AA, Greenberg HB (1996) Protective effect of rotavirus VP6-specific IgA monoclonal antibodies that lack neutralizing activity. Science 272: 104–107. 8600516 10.1126/science.272.5258.104

[24] Feng N, Lawton JA, Gilbert J, Kuklin N, Vo P, et al (2002) Inhibition of rotavirus replication by a non-neutralizing, rotavirus VP6-specific IgA mAb. J Clin Invest 109: 1203–1213. 11994409 10.1172/JCI14397 PMC150959

[25] Corthesy B, Benureau Y, Perrier C, Fourgeux C, Parez N, et al (2006) Rotavirus anti-VP6 secretory immunoglobulin A contributes to protection via intracellular neutralization but not via immune exclusion. J Virol 80: 10692–10699. 16956954 10.1128/JVI.00927-06 PMC1641769

[26] Kallewaard NL, McKinney BA, Gu Y, Chen A, Prasad BV, et al (2008) Functional maturation of the human antibody response to rotavirus. J Immunol 180: 3980–3989. 18322207 10.4049/jimmunol.180.6.3980

[27] McLean GR, Torres M, Elguezabal N, Nakouzi A, Casadevall A (2002) Isotype can affect the fine specificity of an antibody for a polysaccharide antigen. J Immunol 169: 1379–1386. 12133962 10.4049/jimmunol.169.3.1379

[28] Wolbank S, Kunert R, Stiegler G, Katinger H (2003) Characterization of human class-switched polymeric (immunoglobulin M [IgM] and IgA) anti-human immunodeficiency virus type 1 antibodies 2F5 and 2G12. J Virol 77: 4095–4103. 12634368 10.1128/JVI.77.7.4095-4103.2003 PMC150633

[29] Johnson JE, Chiu W (2000) Structures of virus and virus-like particles. Current Opinion in Structural Biology 10: 229–235. 10753814 10.1016/s0959-440x(00)00073-7

[30] Kaufmann B, Nybakken GE, Chipman PR, Zhang W, Diamond MS, et al (2006) West Nile virus in complex with the Fab fragment of a neutralizing monoclonal antibody. Proc Natl Acad Sci U S A 103: 12400–12404. 16895988 10.1073/pnas.0603488103 PMC1567891

[31] Stewart PL, Chiu CY, Huang S, Muir T, Zhao Y, et al (1997) Cryo-EM visualization of an exposed RGD epitope on adenovirus that escapes antibody neutralization. EMBO Journal 16: 1189–1198. 9135136 10.1093/emboj/16.6.1189 PMC1169718

[32] Diprose JM, Burroughs JN, Sutton GC, Goldsmith A, Gouet P, et al (2001) Translocation portals for the substrates and products of a viral transcription complex: the bluetongue virus core. EMBO J 20: 7229–7239. 11742999 10.1093/emboj/20.24.7229 PMC125797

[33] Mathieu M, Petitpas I, Navaza J, Lepault J, Kohli E, et al (2001) Atomic structure of the major capsid protein of rotavirus: implications for the architecture of the virion. EMBO J 20: 1485–1497. 11285213 10.1093/emboj/20.7.1485 PMC145492

[34] McKinney BA, Kallewaard NL, Crowe JE Jr, Meiler J (2007) Using the natural evolution of a rotavirus-specific human monoclonal antibody to predict the complex topography of a viral antigenic site. Immunome Res 3: 8. 17877819 10.1186/1745-7580-3-8 PMC2042970

[35] Fleishman SJ, Leaver-Fay A, Corn JE, Strauch EM, Khare SD, et al (2011) RosettaScripts: a scripting language interface to the Rosetta macromolecular modeling suite. PLoS One 6: e20161. 21731610 10.1371/journal.pone.0020161 PMC3123292

[36] Chen SC, Fynan EF, Robinson HL, Lu S, Greenberg HB, et al (1997) Protective immunity induced by rotavirus DNA vaccines. Vaccine 15: 899–902. 9234543 10.1016/s0264-410x(96)00272-1

[37] Herrmann JE, Chen SC, Fynan EF, Santoro JC, Greenberg HB, et al (1996) Protection against rotavirus infections by DNA vaccination. J Infect Dis 174 Suppl 1: S93–97. 8752297 10.1093/infdis/174.supplement_1.s93

[38] O'Neal CM, Crawford SE, Estes MK, Conner ME (1997) Rotavirus virus-like particles administered mucosally induce protective immunity. J Virol 71: 8707–8717. 9343229 10.1128/jvi.71.11.8707-8717.1997 PMC192335

[39] Thouvenin E, Schoehn G, Rey F, Petitpas I, Mathieu M, et al (2001) Antibody inhibition of the transcriptase activity of the rotavirus DLP: a structural view. J Mol Biol 307: 161–172. 11243811 10.1006/jmbi.2000.4479

[40] Lawton JA, Estes MK, Prasad BV (1999) Comparative structural analysis of transcriptionally competent and incompetent rotavirus-antibody complexes. Proceedings of the National Academy of Sciences of the United States of America 96: 5428–5433. 10318900 10.1073/pnas.96.10.5428 PMC21876

[41] Durocher Y, Perret S, Kamen A (2002) High-level and high-throughput recombinant protein production by transient transfection of suspension-growing human 293-EBNA1 cells. Nucleic Acids Research 30: E9. 11788735 10.1093/nar/30.2.e9 PMC99848

[42] McLean GR, Nakouzi A, Casadevall A, Green NS (2000) Human and murine immunoglobulin expression vector cassettes. Mol Immunol 37: 837–845. 11257305 10.1016/s0161-5890(00)00101-2

[43] Winter G (2010) xia2: an expert system for macromolecular crystallography data reduction. Journal of applied crystallography 43: 186–190.

[44] Sauter NK, Grosse-Kunstleve RW, Adams PD (2004) Robust indexing for automatic data collection. Journal of applied crystallography 37: 399–409. 20090869 10.1107/S0021889804005874 PMC2808709

[45] Kabsch W (2010) Xds. Acta Crystallographica Section D: Biological Crystallography 66: 125–132. 20124692 10.1107/S0907444909047337 PMC2815665

[46] Kabsch W (2010) Integration, scaling, space-group assignment and post-refinement. Acta Crystallographica Section D: Biological Crystallography 66: 133–144. 20124693 10.1107/S0907444909047374 PMC2815666

[47] Evans P (2006) Scaling and assessment of data quality. Acta Crystallographica Section D: Biological Crystallography 62: 72–82. 16369096 10.1107/S0907444905036693

[48] Brunger AT (1997) Free R value: cross-validation in crystallography. Methods Enzymol 277: 366–396. 18488318 10.1016/s0076-6879(97)77021-6

[49] Vagin A, Teplyakov A (1997) MOLREP: an automated program for molecular replacement. Journal of applied crystallography 30: 1022–1025.

[50] Adams PD, Grosse-Kunstleve RW, Hung LW, Ioerger TR, McCoy AJ, et al (2002) PHENIX: building new software for automated crystallographic structure determination. Acta Crystallographica Section D: Biological Crystallography 58: 1948–1954. 12393927 10.1107/s0907444902016657

[51] Emsley P, Cowtan K (2004) Coot: model-building tools for molecular graphics. Acta Crystallographica Section D: Biological Crystallography 60: 2126–2132. 15572765 10.1107/S0907444904019158

[52] Brünger AT, Adams PD, Rice LM (1997) New applications of simulated annealing in X-ray crystallography and solution NMR. Structure 5: 325–336. 9083112 10.1016/s0969-2126(97)00190-1

[53] Painter J, Merritt EA (2006) Optimal description of a protein structure in terms of multiple groups undergoing TLS motion. Acta Crystallographica Section D: Biological Crystallography 62: 439–450. 16552146 10.1107/S0907444906005270

[54] Schwarz BA, Bange R, Vahlenkamp TW, Johne R, Muller H (2002) Detection and quantitation of group A rotaviruses by competitive and real-time reverse transcription-polymerase chain reaction. J Virol Methods 105: 277–285. 12270660 10.1016/s0166-0934(02)00118-0

[55] Li S, Tsalkova T, White MA, Mei FC, Liu T, et al (2011) Mechanism of intracellular cAMP sensor Epac2 activation: cAMP-induced conformational changes identified by amide hydrogen/deuterium exchange mass spectrometry (DXMS). J Biol Chem 286: 17889–17897. 21454623 10.1074/jbc.M111.224535 PMC3093864

[56] Hsu S, Kim Y, Li S, Durrant ES, Pace RM, et al (2009) Structural insights into glucan phosphatase dynamics using amide hydrogen-deuterium exchange mass spectrometry. Biochemistry 48: 9891–9902. 19754155 10.1021/bi9008853 PMC2767375

[57] Lu WD, Liu T, Li S, Woods VL Jr, Hook V (2012) The prohormone proenkephalin possesses differential conformational features of subdomains revealed by rapid H-D exchange mass spectrometry. Protein Sci 21: 178–187. 22102294 10.1002/pro.2000 PMC3324762

[58] Zhang Z, Smith DL (1993) Determination of amide hydrogen exchange by mass spectrometry: a new tool for protein structure elucidation. Protein Sci 2: 522–531. 8390883 10.1002/pro.5560020404 PMC2142359

