# Supplementary material for: Human Rotavirus VP6-Specific Antibodies Mediate Intracellular Neutralization by Binding to a Quaternary Structure in the Transcriptional Pore
Source: PLoS One. 2013 May 9;8(5):e61101. doi: 10.1371/journal.pone.0061101 (PMC3650007; doi:10.1371/journal.pone.0061101)
Supplement: Figure S1 — Alignment of group A and non-group A rotavirus VP6 sequences showing conservation of the negatively-charged surface electrostatic potential in the VP6 trimer. Alignment of representative sequences from group A and non-group A rotavirus VP6 sequences (specific strains are shown in table below) was performed using ClustalW2, the multiple sequence alignment computer program. Numbers at the top indicate the residue number. Small letters represent 1-letter residue abbreviations of the consensus VP6 sequence based on the aligned representative VP6 sequences. The gray bar graphs represent identity histograms showing conservation levels of individual residues at each position. The dashes within the sequences indicate absence of residues from the specific strain sequences accessed from GenBank. Residues below the solid black line indicate those that are shown to contribute to the negatively-charged electrostatic patch within the transcriptional pore formed by VP6 trimers. (PDF) [file pone.0061101.s001.pdf]

```
Residue #                          1         11        21        31        41        51        61        71        81        91
Consensus                          mevlysls---ktlkdardkivegtlysnvsdliqqfnqmivtmngndfqtggignlpirnwtfdfgllgttllnldanyvenarttieyfidfidavcm
Conservation
RV Group A [ subgroup I ]          MDVLYSLS---KTLKDARDKIVEGTLYSNVSDLIQQFNQMIITMNGNEFQTGGIGNLPIRNWNFDFGLLGTTLLNLDANYVETARNTIDYFVDFVDNVCM
RV Group A [ subgroup II ]         MEVLYSLS---KTLKDARDKIVEGTLYSNVSDLIQQFNQMIVTMNGNDFQTGGIGNLPVRNWTFDFGLLGTTLLNLDANYVENARTTIEYFIDFIDNVCM
RV Group A [ non-subgroup I & II ] MEVLYSLS---KTLKDARDKIVEGTLYSNVSDLIQQFNQMIVTMNGNDFQTGGIGNLPIRNWTFDFGLLGTTLLNLDANYVETARTTIEYFIDFIDNVCM
RV Group B                         MDLIETVNACVKLQKRVLGLAPNTNLNTAGQSVLNDYNALASRVNGKTYALLNQI-AVYTPYTINAPIISLAVR-ISTDDYDDMRSGIESILDVLAAAIR
RV Group C                         MEVLYSLS---KTLKDARDKIVEGTLYSNVSDLIQQFNQMIVTMNGNDFQTGGIGNLPIRNWTFDFGLLGTTLLNLDANYVENARTTIEYFIDFIDNVCM
RV Group D                         MEALSSIA---LTVREAREKIINGTIYSNVSDVIQQFNQMVRVLNGSTFTTGGLATMPLREWTFDLPQLGTTLLNIDANYVESMTPTLDMLTEFVIAVCE
RV Group F                         MEAIYSIA---RTIADAEEKIVVGTLFSNVQQIVMQMNEVIKVMNGSQFLTGGVGNMGVTQWTFNHGQLGVAPLYVDENYREELMQTINYYKRFVHAVCE
RV Group G                         MDLIETVNAVVDLQKRIYELSSNTNLSSKGQQVVNDYNALASRVNGKTYALHDQT-AVLTPYIINMNLISISTR-ISTDDYEAMKEGMNGLLDVIAAAIR

Residue #                          101       111       121       131       141       151       161       171       181       191
Consensus                          demaresqrngia-pqsealrklsgikfkrinfdnsseyienwnlqnrrqrtg--------------fvfhkpnifpysasitlnrsqpahdnlmgtrwl
Conservation
RV Group A [ subgroup I ]          DEMVRESQRNGIA-PQSDSLRKLSGIKFKRINFDNSSEYIENWNLQNRRQRTG--------------FTFHKPNIFPYSASFTLNRSQPAHDNLMGTMWL
RV Group A [ subgroup II ]         DEMARESQRNGVA-PQSEALRKLAGIKFKRINFDNSSEYIENWNLQNRRQRTG--------------FVFHKPNIFPYSASFTLNRSQPMHDNLMGTMWL
RV Group A [ non-subgroup I & II ] DEMARESQRNGVA-PQSEALRKLAGIKFKRINFNNSSEYIENWNLQNRRQRTG--------------FVFHKPNIFPYSASFTLNRSQPMHDNLMGTMWL
RV Group B                         TEGSRPTRVIERRVLEPNVKQLIDDLRLKSLVSDLSIANLAAVDT--AMIQPEVIETENPLYADIIEQIVHRPNIGMTGGNIRAT----------LGRWS
RV Group C                         DEIARESQRNGIA-PQSEALRKLSGIKFKRINFDNSSDYIENWNLQNRRQRTG--------------FVFHKPNILPYSASFTLNRSQPAHDNLMGTMWI
RV Group D                         TELLVDNNRNGAY-PQSEALRLLSNNKYVFLNMDLGSKYISEWHYRLSARDPM--------------FSNHVPYIFPYDMAIAYDRVTAAYDNVSGTRFA
RV Group F                         TEMCLESQRGGAT-MQSAAMQALQTVKFKNLSYDYSENYIKQWALQKVRVRTA--------------YSSRNPYVVPELNTITYRRSQAAMDNVAGTRVM
RV Group G                         TECSRRVRAIEQRVLEPVVKMLIEDIRLRAQVSKIAIANMSAYDT--AKLEPQIVNIENPLRPGIVDDVLQRPNFTQRGGGIRYT----------TGRWS

Residue #                          201       211       221       231       241       251       261       271       281       291
Consensus                          n-agseiqvagfdysca-napaniqqfehivqlrraltttatitllpdaerfsfprvinsadgattwffnpvilrpn--nvevefllngqiintyqarfgt
Conservation
RV Group A [ subgroup I ]          N-AGSEIQVAGFDYSCAINAPANTQQFEHIVQLRRVLTTATITLLPDAERFSFPRVINSADGATTWYFNPVILRPN--NVEVEFLLNGQIINTYQARFGT
RV Group A [ subgroup II ]         N-AGSEIQVAGFDYSCAINAPANIQQFEHIVQLRRALTTATITLLPDAERFSFPRVINSADGATTWFFNPVILRPN--NVEVEFLLNGQIINTYQARFGT
RV Group A [ non-subgroup I & II ] N-AGSEIQVAGFDYSCALNAPANIQQFEHIVQLRRALTTATITLLPDAERFSFPRVINSADGATTWFFNPIILRPN--NVEVEFLLNGQIINTYQARFGT
RV Group B                         GNKGVVTCMSGMD-----------SEHRFTVELKTRTCGIINIVYIPTSGTILIPMPAGR--NREGDLIDVSAEMMADDFAIDFMDDDNIIQTE-TGVGV
RV Group C                         N-AGSEIQVAGFDYSCAFNAPANIQQFEHVVPLRRALTTATITLLPDAERFSFPRVINSADGTTTWYFNPVILRPS--NVEVEFLLNGQIINTYQARFGT
RV Group D                         S-LNNAIHFAAFDQDFVRGQPANARQFEYLYNLRTPVSNATIVIHPISDILSVPSMIRNQAATHYWPYNPYNIPTFRDDIRVEFQLAGQVIYVA-ANLGM
RV Group F                         A-IGDTIKFAVFDQDFARTEPGQMWESNFEYNMPVPISDATIQVGTDASGLLNDQVLITNTYNTTWLYVPFQVPLT--RFTIEFYNTGNLVYQIVA-PGM
RV Group G                         GNKGIVTCVSGTD-----------GVHTIEIQLRTTLTGILNLLYVPAPGKIVQSRGRAQGVPVTLRCSDVSPDMARGDIVIEFLRNGAVVDAE-GGSGT

Residue #                          301       311       321       331       341       351       361       371       381       391
Consensus                          iiarnfdtirlsfqlmrppn-----mtpavanlfpqaqpf-hhatvg-ltlriesavcesvlad----anetlyanvtavrqeyaipvgpvfppgmnwte
Conservation
RV Group A [ subgroup I ]          IIARNFDTIRLSFQLMRPPN-----MTPTVAALFPNAQPFEHHATVG-LTLRIESAVCESVLAD----ASETMLANVTSVRQEYAIPVGPVFPPGMNWTD
RV Group A [ subgroup II ]         IIARNFDTIRFIFQLMRPPN-----MTPAVNALLPQAQPFQHHATVG-LTLRIESAVCESVLAD----ANETLLANVTAVRQEYAIPVGPIFPPDMNWTE
RV Group A [ non-subgroup I & II ] IVARNFDTIRLSFQLMRPPN-----MTPAVNALFPQAQPFQHHATVG-LTLRIESAVCESVLAD----ANETLLANVTAVRQEYAIPVGPVFPPGMNWTE
RV Group B                         YSFPMCNRIRFRINPWNNQKDDDNLGTVHMVNWAQGTAP---KQPAISFMFETRRTFTEGDYQHLSRCAPKAQYMMDTQ-----FNDVSFTNRPAVDWNI
RV Group C                         IIARNFDTIRLSFQLVRPPN-----MTPAVANLFPQAPPFIFHATVG-LTLRIESAVCESVLAD----ASETLLANVTAVRQEYAIPVGPVFPPGMNWTE
RV Group D                         HTIPQFDAVNIILTMRRLPL------LADLQNIFPAGNPSATHQAVISTKIEVLNATTETTVPS----INEHLYALIVGTRGRYQMQAGPVFPPGMRWDD
RV Group F                         HCVPTFNSIIFRLRIVRPAV-----LSQQLQQYFPAQNPGNVHATVV-ISLQIKSCVTDVVLAD----PDSTHYARITNVRTLYGIPVGPVFPPNMAWDT
RV Group G                         FQFTQCDTIRIRVEPWSIVKNLN--PTPDFANWNQNQAN---SQPTVLIMFEIRQAYTQLENDEMSADAPKVQYYLDTI-----FTQDSFVRHPNVLWRT
```

Figure S1. Alignment of group A and non-group A rotavirus VP6 sequences showing conservation of the negatively-charged surface electrostatic potential in the VP6 trimer. Alignment of representative sequences from group A and non-group A rotavirus VP6 sequences (specific strains are shown in table below) was performed using ClustalW2, the multiple sequence alignement computer program. Numbers at the top indicate the residue number. Small letters represent 1-letter residue abbreviations of the consensus VP6 sequence based on the aligned representative VP6 sequences. The gray bar graphs represent identity histograms showing conservation levels of individual residues at each position. The dashes within the sequences indicate absence of residues from the specific strain sequences accessed from Genbank. Residues below the solid black line indicate those that are shown to contribute to the negatively-charged electrostatic patch within the VP6 trimers.
