# Supplementary material for: Human Rotavirus VP6-Specific Antibodies Mediate Intracellular Neutralization by Binding to a Quaternary Structure in the Transcriptional Pore
Source: PLoS One. 2013 May 9;8(5):e61101. doi: 10.1371/journal.pone.0061101 (PMC3650007; doi:10.1371/journal.pone.0061101)
Supplement: Table S1 — , Sources of the VP6 sequences used for alignment analysis of conserved negative patch. Table S2, Data collection and refinement statistics. (PDF) [file pone.0061101.s002.pdf]

Table S1: Sources of the VP6 sequences used for alignment analysis of conserved negative patch

Rotavirus Group	Strain	Genbank Accension
Group A (subgroup I)	Human rotavirus A/1076	BAA00237
Group A (subgroup II)	Human rotavirus A	CAA41010
Group A (non-subgroup I & II)	Human rotavirus A/CMH185/01	ABY82714
Group B	Human rotavirus group B/India	BAB20454
Group C	Porcine rotavirus/WH-a/2010	AER25319
Group D	Rotavirus D chicken/05V0049/DEU/2005	ADN06428
Group F	Rotavirus F chicken/03V0568/DEU/2003	ADZ44637
Group G	Rotavirus G chicken/03V0567/DEU/2003	ADZ44638
