# Supplementary material for: Human Rotavirus VP6-Specific Antibodies Mediate Intracellular Neutralization by Binding to a Quaternary Structure in the Transcriptional Pore
Source: PLoS One. 2013 May 9;8(5):e61101. doi: 10.1371/journal.pone.0061101 (PMC3650007; doi:10.1371/journal.pone.0061101)
Supplement: Figure S2 — RV6-26 Fab paratope as determined by deuterium exchange mass spectroscopy. (A) Side view of the predicted paratope regions of RV6-26 Fab on the antibody structure (PDB-ID 4HFW). The color scheme is as used in Figure 6. The antibody light chain is represented in pink and the heavy chain is shown in pale green. Red and blue depict the antibody regions predicted to form the RV6-26 Fab paratope in the light and heavy chains respectively. (B) The top view of the RV6-26 Fab showing the antigen combining region with the DXMS-predicted regions shown to be involved in antigen interactions represented in red and blue on the light and heavy chains, respectively. (PDF) [file pone.0061101.s003.pdf]

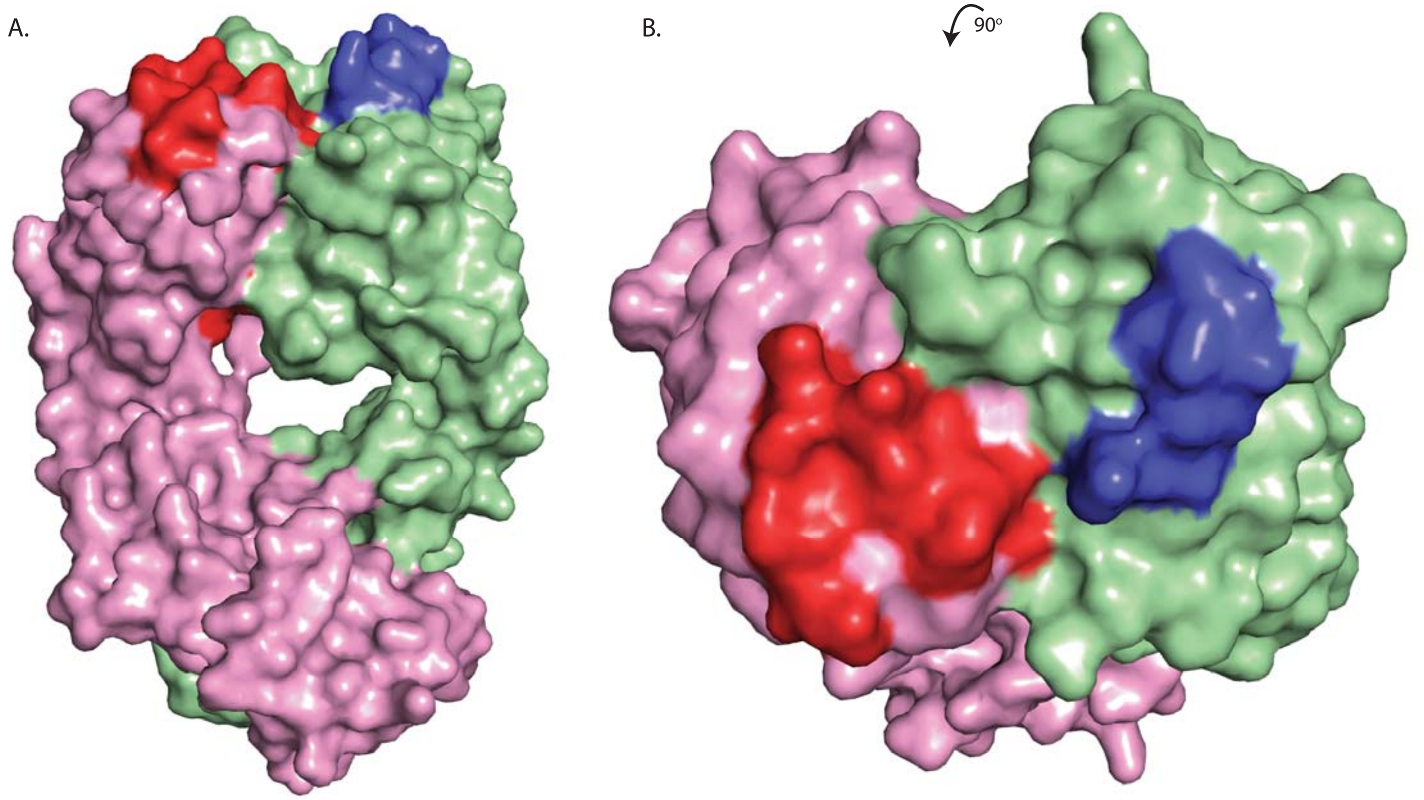

Figure S2. RV6-26 Fab paratope as determined by deuterium exchange mass spectroscopy . (A) Side view of the predicted paratope regions of RV6-26 Fab on the antibody structure (PDB-ID 4HFW). The color scheme is as used in Figure 6. The antibody light chain is represented in pink and the heavy chain is shown in pale green. Red and blue depict the antibody regions predicted to form the RV6-26 Fab paratope in the light and heavy chains respectively. (B) The top view of the RV6-26 Fab showing the antigen combining region with the DXMS-predicted regions shown to be involved in antigen interactions represented in red and blue on the light and heavy chains, respectively.
