# Supplementary material for: Human Rotavirus VP6-Specific Antibodies Mediate Intracellular Neutralization by Binding to a Quaternary Structure in the Transcriptional Pore
Source: PLoS One. 2013 May 9;8(5):e61101. doi: 10.1371/journal.pone.0061101 (PMC3650007; doi:10.1371/journal.pone.0061101)
Supplement: Table S2 — Data collection and refinement statistics. (PDF) [file pone.0061101.s004.pdf]

Table S2. Data collection and refinement statistics

**Data collection**	
Space group	$P2_1$
Cell parameters	a=68.10
	b=97.92
	c=94.23
	β=108.9
Wavelength	0.97850
Resolution (Å)	50-2.6
Completeness (%)	50-2.25 Å: 99.7 (100)
Redundancy	50-2.6Å: 4.2(4.2)
I/sigmaI	50-2.6 Å: 8.5(2.2)
**Refinement**	
	50-2.6 Å
Resolution (Å)	
No. reflections	35,513
Working set	
No. reflections free set	1,778
R_{work} / R_{free} %	20.07/23.50
R.m.s. deviations	
Bond lengths (Å)	0.004
Bond angles (°)	1.108
Ramachandran Plot	Favored=98.1%
	Allowed=1.65%
	Outliers=0.24%
